# Supplementary material for: Genomic insights into the evolution of Echinochloa species as weed and orphan crop
Source: Nat Commun. 2022 Feb 3;13:689. doi: 10.1038/s41467-022-28359-9 (PMC8814039; doi:10.1038/s41467-022-28359-9)
Supplement: Supplementary file 1 — Supplementary Information [file 41467_2022_28359_MOESM1_ESM.pdf]

Genomic insights into the evolution of *Echinochloa* species as weed and orphan crop

Wu *et al.*

Supplementary Table 1. Likelihood comparison of demographic models for divergence history among varieties of the *E. crus-galli* complex. See more in Supplementary Fig. 24.

Scenario	# Params	Delta likelihood	AIC
Model 1	25	50472.854	3852146.871
Model 2	25	50716.545	3853269.109
Model 3	26	49627.019	3848253.657
Model 4	26	47220.113	3837169.445
Model 5	27	46882.136	3835615.003

Supplementary Table 2. Best-fit scenario of gene flow between groups. Schematic illustration of the best scenario is shown in Fig. 2e. See more in Supplementary Fig. 25.

**Pop1**	**Pop2**	**Best scenario**
CUL	ECCG	Recent_ancient21
CUL	ECCP	Recent_ancient21
ECCG	ECCP	Recent_ancient12
CUL	ECOR	Ancient_recent12
ECCG	ECOR	Recent_ancient12
ECCP	ECOR	Recent_geneflow
CUL	ECPR	Recent_ancient21
ECCG	ECPR	Recent_geneflow
ECCP	ECPR	Recent_geneflow
ECOR	ECPR	Recent_geneflow
CUL	EOYR	Recent_ancient21
ECCG	EOYR	Recent_geneflow
ECCP	EOYR	Recent_geneflow
ECOR	EOYR	Recent_geneflow
ECPR	EOYR	Recent_ancient21

Supplementary Table 3. RNA-seq datasets used for *Echinochloa* genome annotation

Species	Tissue	Treatment	SRA
E. crus-galli	seedling	/	SRR5903813-SRR5903830
	leaf	/	CRR143187-CRR143189
	leaf	blast infection	CRR143190-CRR143192
	leaf	drought	CRR143193-CRR143195
	root	/	CRR143196-CRR143198
	root	drought	CRR143199-CRR143201
E. colona	leaf	/	CRR292857-CRR292859
	panicle	/	CRR292860-CRR292862
E. oryzicola	seedling	/	CRR143148
	leaf	/	CRR143172-CRR143174
	leaf	blast infection	CRR143175-CRR143177
	leaf	drought	CRR143178-CRR143180
	root	/	CRR143181-CRR143183
	root	drought	CRR143184-CRR143186

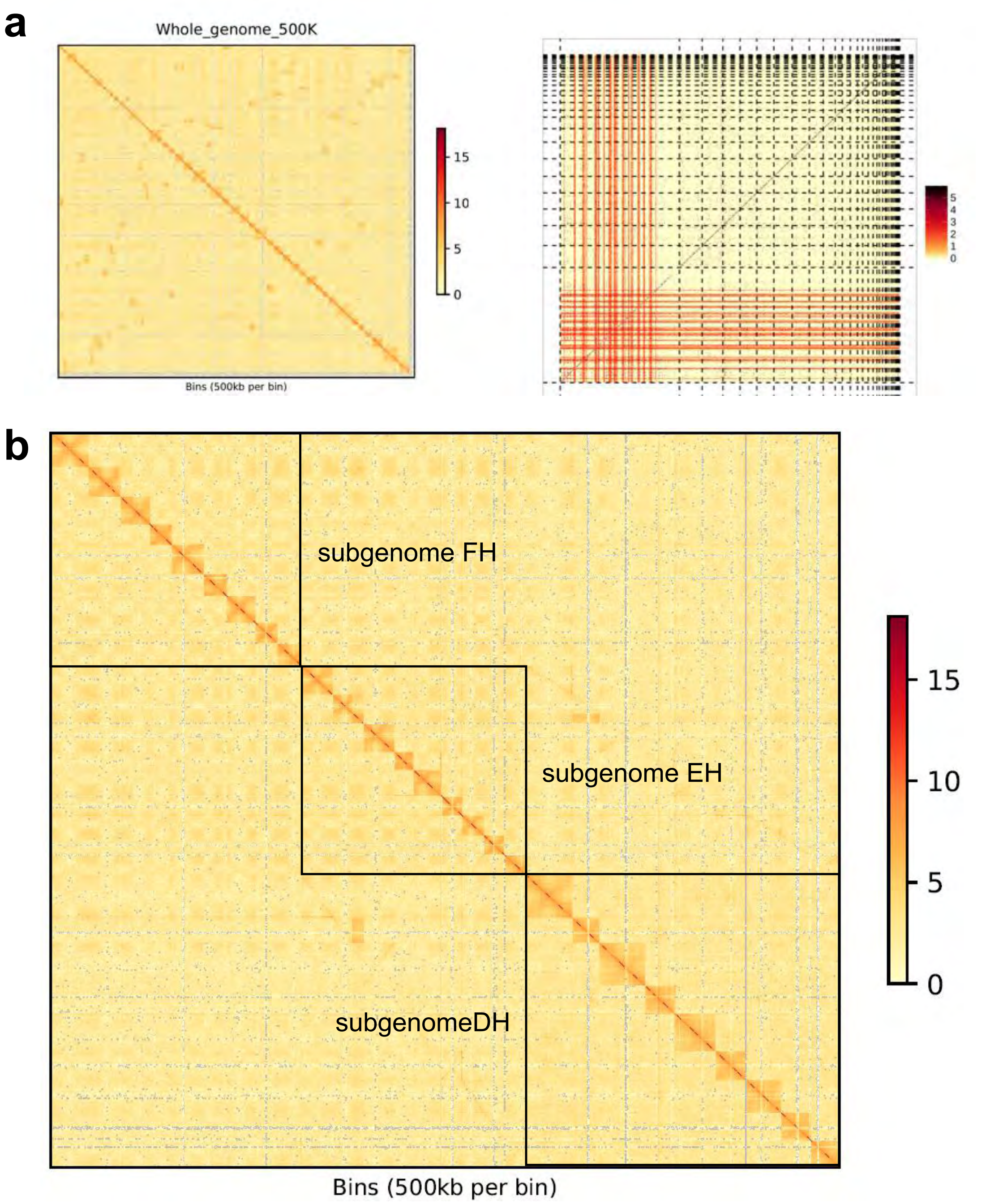

Supplementary Figure 1. Genome-wide HiC interaction heatmap.
a Interaction heatmap based on scaffolding assemblies of *E. colona* using ALLHiC with diploid *E. haploclada* to construct allle.ctg.table (left) and Lachesis (right). **b** Hi-C interaction heatmap based on DipHiC assembly of *E.colona* genome. 27 chromosomes are assigned in subgenomes DH, EH and FH, respectively.

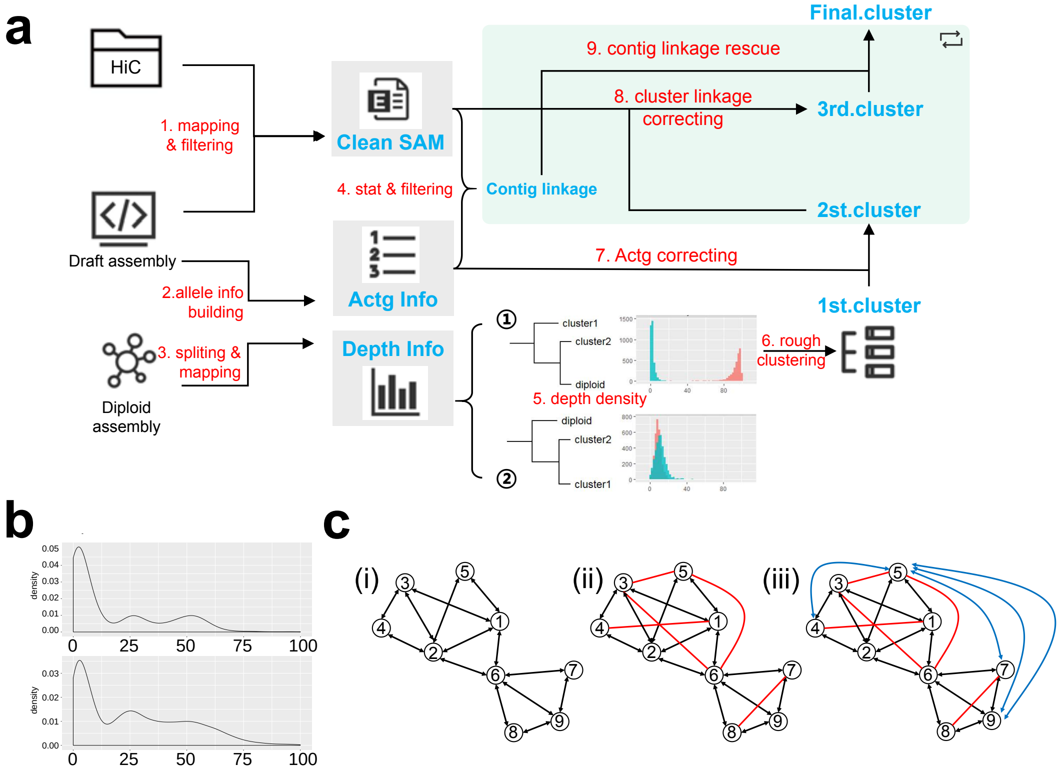

#Contig	#depth	#1st	#2nd	#2nd note	#links to cluster1	#links to cluster2	#links to cluster3	
ptg000001l	45.7	uncluster	cluster3	pass	280480	236798	532062	
ptg000002l	5.0	cluster1	cluster1	pass	473084	281574	353982	
ptg000003l	44.3	uncluster	uncluster	uncluster		550509	438942	825946
ptg000004l	60.5	cluster3	cluster3	pass	710871	563809	1230047	
ptg000005l	52.7	cluster3	cluster3	pass	734054	577643	1065066	
ptg000006l	3.8	cluster1	cluster1	pass	643291	349132	425820	
ptg000007l	3.8	cluster1	cluster1	pass	608674	309968	375596	
ptg000008l	51.7	cluster3	cluster3	pass	476129	368177	693272	
ptg000009l	31.7	cluster2	cluster2	pass	67857	128968	61339	
ptg000010l	4.9	cluster1	cluster1	pass	593720	414556	450686	
ptg000011l	71.8	cluster3	cluster3	pass	315397	245495	634153	
ptg000012l	53.4	cluster3	cluster3	pass	163204	142566	330320	
ptg000013l	38.4	uncluster	uncluster	uncluster		812379	806151	849014
ptg000014l	47.7	cluster3	cluster3	pass	504294	412294	950810	
ptg000015l	3.7	cluster1	cluster1	pass	704167	380049	440177	
ptg000016l	55.4	cluster3	cluster3	pass	177030	145078	385386	
ptg000017l	7.9	cluster1	cluster1	pass	515265	291099	317902	
ptg000018l	31.0	cluster2	cluster2	pass	386702	422271	337847	
ptg000019l	7.2	cluster1	cluster1	pass	311608	146586	175190	
ptg000020l	26.1	cluster2	cluster2	pass	725336	613369	658723	
ptg000021l	0.9	cluster1	uncluster	noActg	2371	1818	3046	
ptg000022l	25.2	cluster2	cluster2	pass	528570	447381	507656	

Supplementary Figure 2. Subgenome distinguishing in hexaploid *E. colona* genome assembly.
a Workflow of subgenome distinguishing. 1, mapping and filtering as regular Hi-C data processing; 2, allele information building based on contig allelism; 3, splitting diploid genome into K-mer and mapping to polyploid draft assembly; 4, counting the links or interactions among contigs; 5, depth distribution; 6, first round of clustering based on depth; 7, second clustering based on allelic contig information; 8, third round clustering based on interactions or linkages; 9, more rounds of clustering based on linkages. **b** Mapping depth distribution when 100-mers split from diploid *E. haploclada* genome are mapped to *E. colona* contigs. The depths are calculated per 1 Mb (upper panel) and 100 kb (lower panel) along *E. colona* contigs. Three peaks are observed clearly, due to the hexaploidy of *E. colona*. **c** Allelic contig information construction in hexaploid. (i) mutually exclusive relationship inferred from large synteny. (ii) two contigs which are mutually exclusive with two exclusive contigs, are from a shared subgenome. (iii) If one contig is exclusive with another contig, then it is exclusive with contigs from the subgenome another contig is from. **d** Examples illustrating three rounds of clustering in *E. colona* subgenome distinguishing. See details in **Supplementary Note 2**.

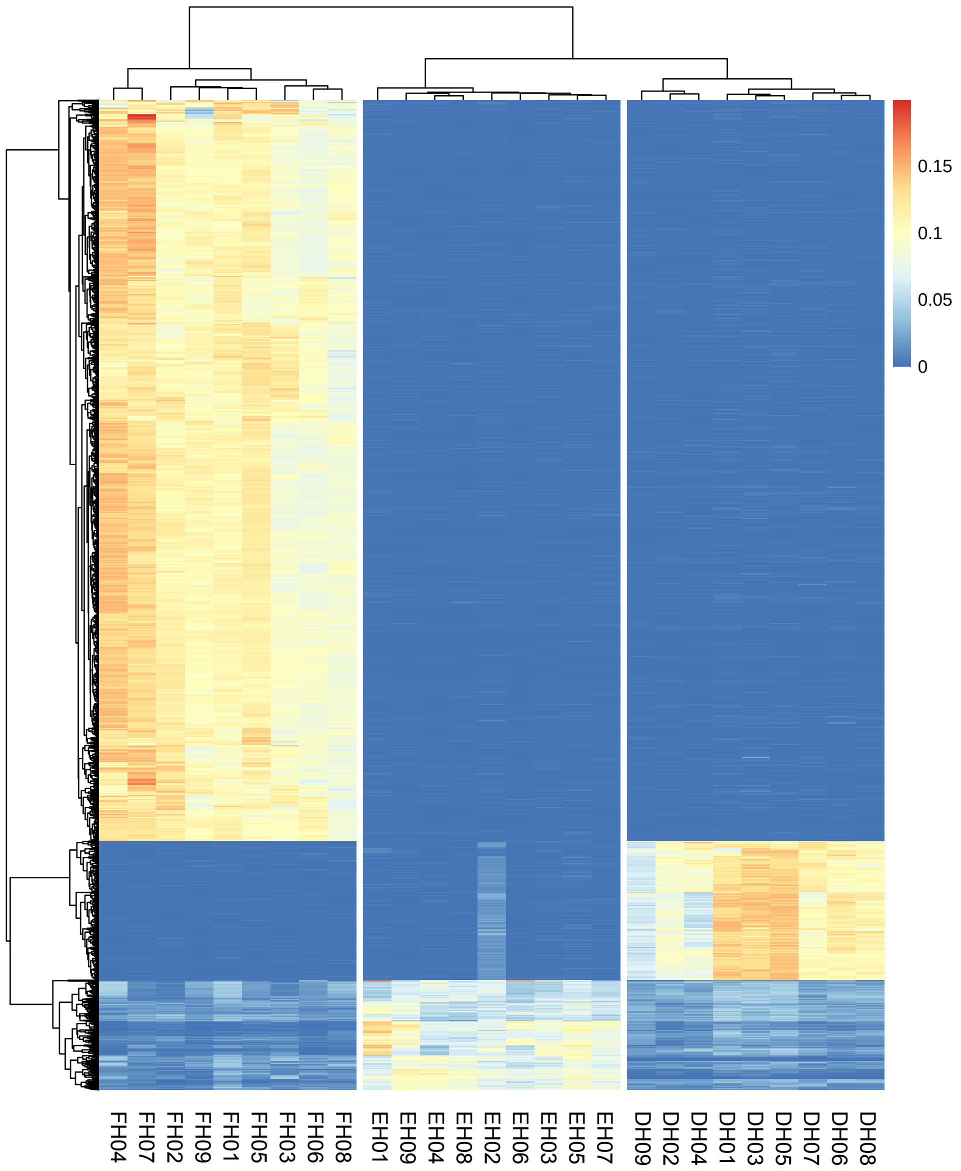

Supplementary Figure 3. The validation of subgenome distinguishing by *K*-mer when *K* = 13. Clustering of counts of 13-mers that differentiate homeologous chromosomes enables the consistent partitioning of the genome into subgenomes in hexaploid *E. colona*. The values are the proportions of *K*-mer copy numbers on each chromosome within the whole genome.

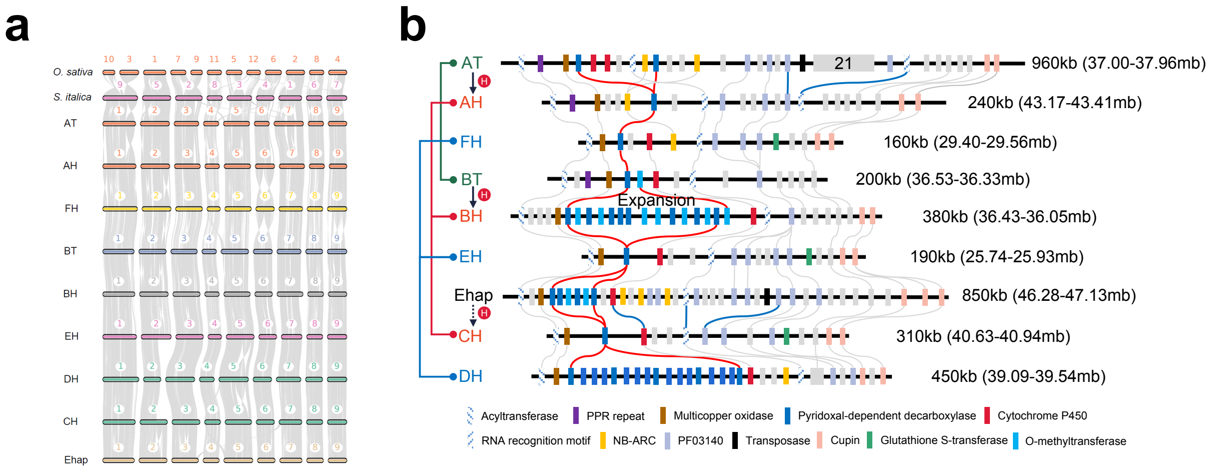

Supplementary Figure 4. Macro and micro genomic synteny among *Echinochloa* subgenomes.

a Macro-synteny among *Echinochloa* subgenomes, *O. sativa* and *S. italica*. **b** Micro-synteny of terminal regions on chromosomes 4 in nine *Echinochloa* subgenomes. The evolutionary relationship is shown at the left. The genes with various functions are marked by different colors. The red lines highlight the local genomic expansion and contraction.

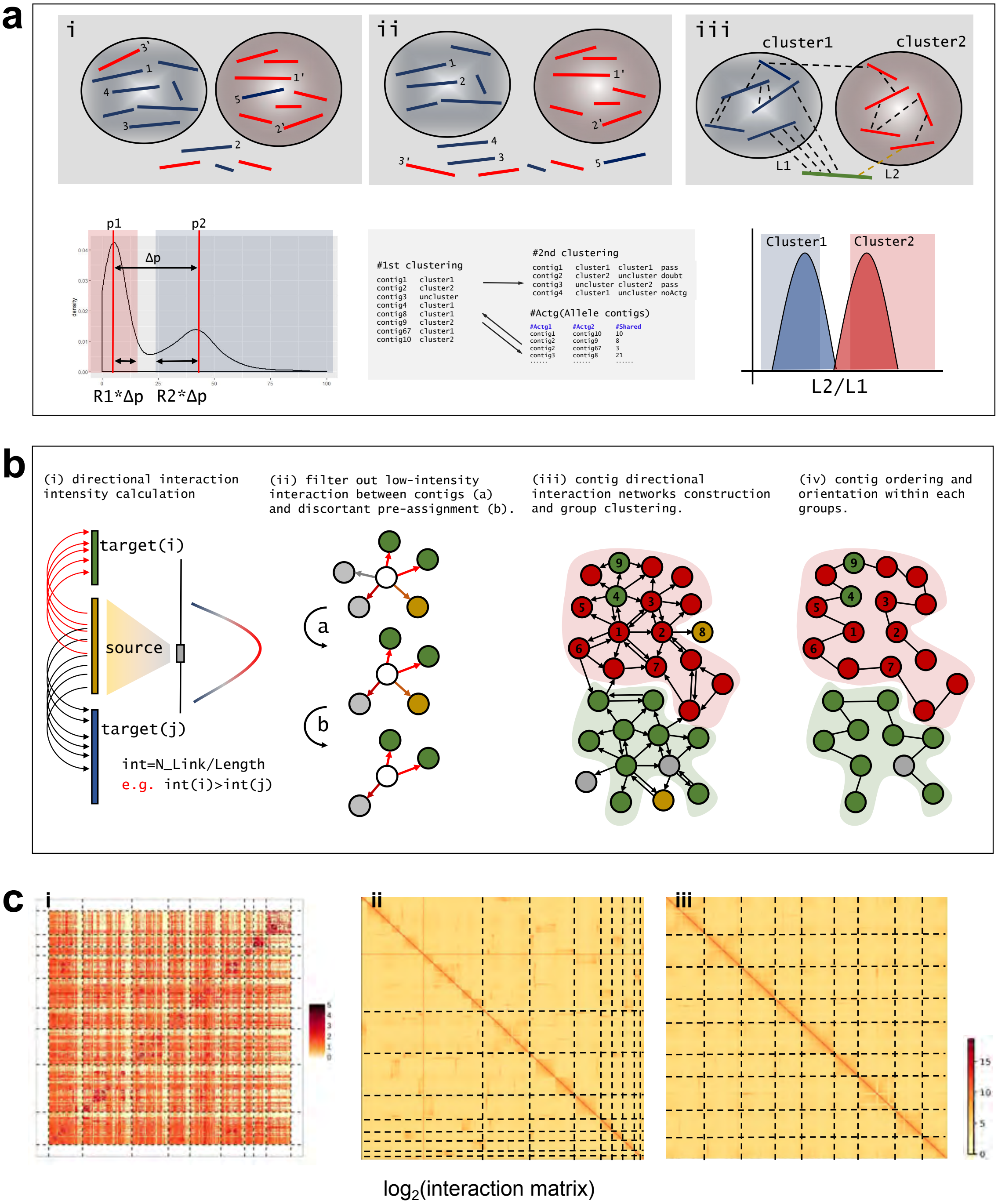

Supplementary Figure 5. Subgenome distinguishing and chromosome building in tetraploid *E. oryzicola* genome assembly.

a Work flow of DipHiC subgenome distinguishing in tetraploid. (i) clustering based on *K*-mer mapping depth. (ii) clustering based on allelic contigs information. (iii) clustering based on interaction linkages. **b** Work flow of DipHiC chromosome building. **c** Performance of Lachesis (i) , AllHiC (ii) and DipHiC (iii) in chromosome building of *cluster1*. See details in **Supplementary Note 3**.

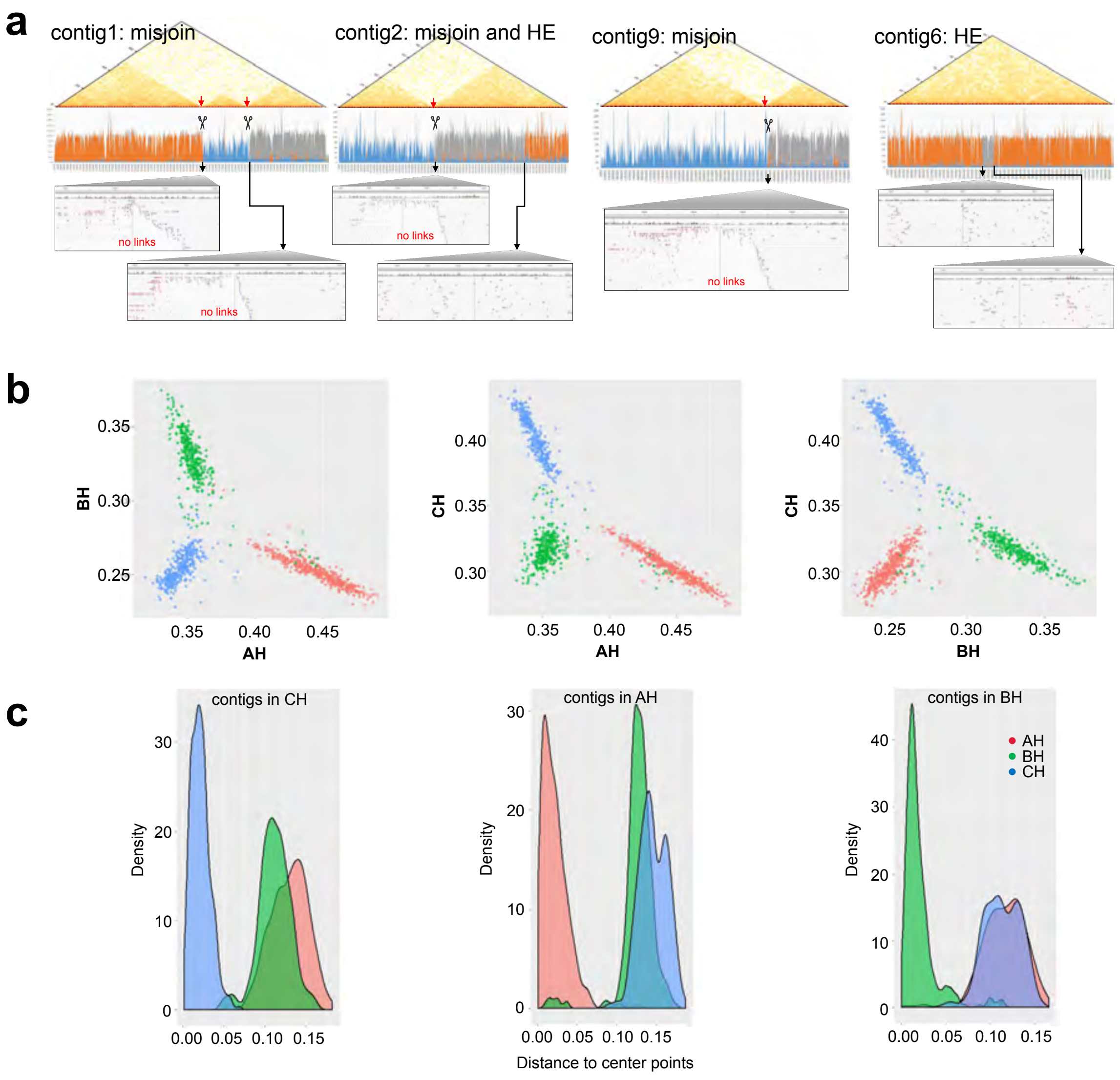

Supplementary Figure 6. Contig correction and subgenome distinguishing in hexaploid *E. crus-galli* genome assembly.

a Examples of contigs with misjoin or homeologous exchanges (HEs). Top panel: Hi-C interaction heat-map within each contig. Bottom panel: mapping depth by three ancestral subgenomes (*E. haploclada* and two subgenomes of *E. oryzicola*) (as indicated by three colors). Bottom panel: IGV screenshots of effective links around junctions when 20-kb mate-paired library reads are mapped. Clippers represent the split junctions before following scaffolding. **b** Proportion of links in a subgenome to each contig. Assuming that contig1 is categorized in cluster AH, then links between contig1 and other contigs are assigned as three parts, links between contig1 and AH, BH and CH contigs. As expected, the proportion of links between contig1 and contigs from AH would be the largest, as shown in red dots. **c** Distance to three cluster center points is used to assign unclustered contigs and to correct clustered contig categories. For example, contig2 is unclustered, we calculate the proportion of its links to other contigs, and euclidean distance is used to measure the possibility of cluster assignment. If the distance to AH center point is significantly shorter than those to BH and CH center points, contig2 would be probably categorized in AH cluster. See details in **Supplementary Note 4**.

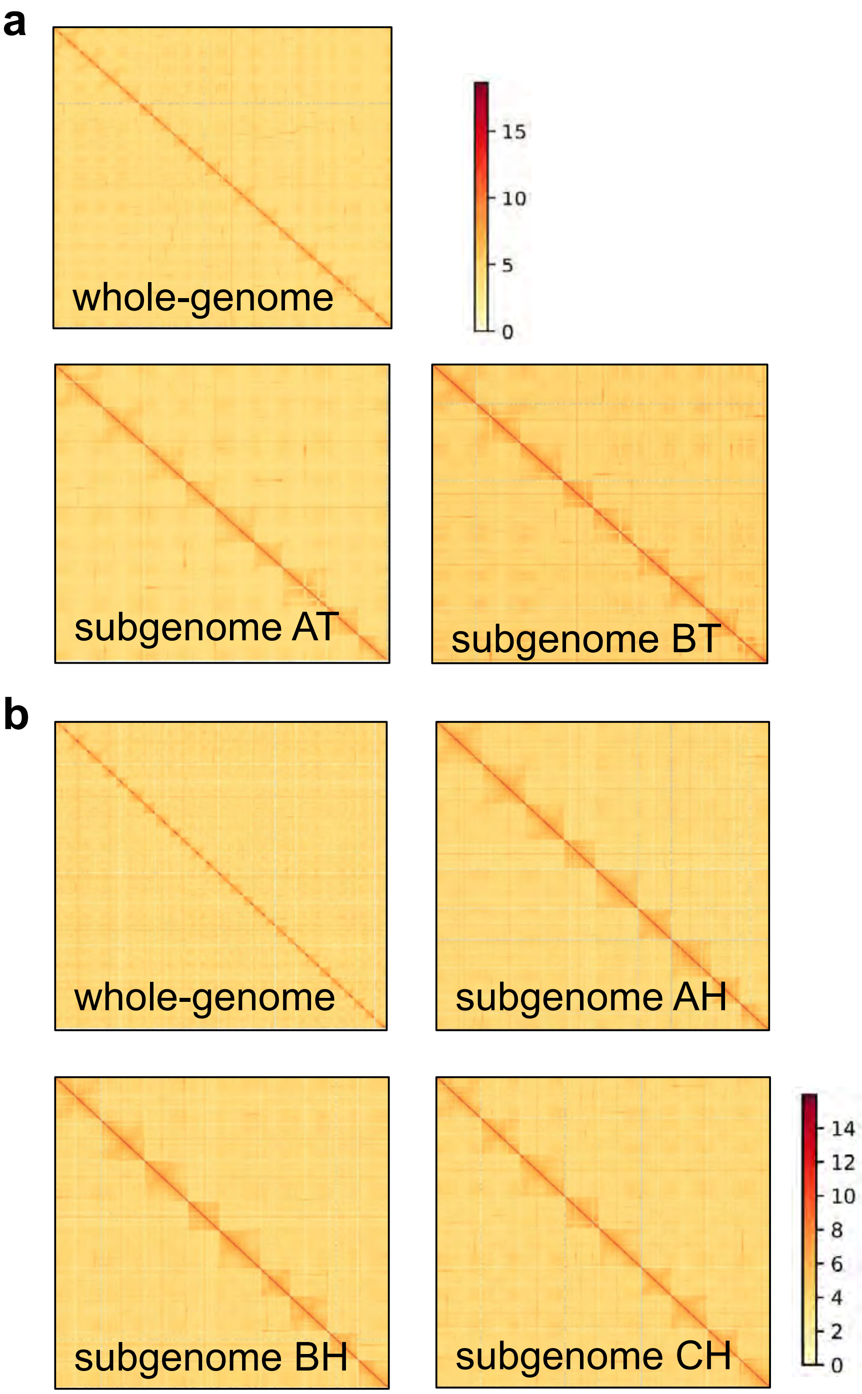

Supplementary Figure 7. Genome-wide HiC all-to-all interaction heatmap in *E. oryzicola* (a) and *E. crus-galli* (b) (sub)genome (every 500 kb).

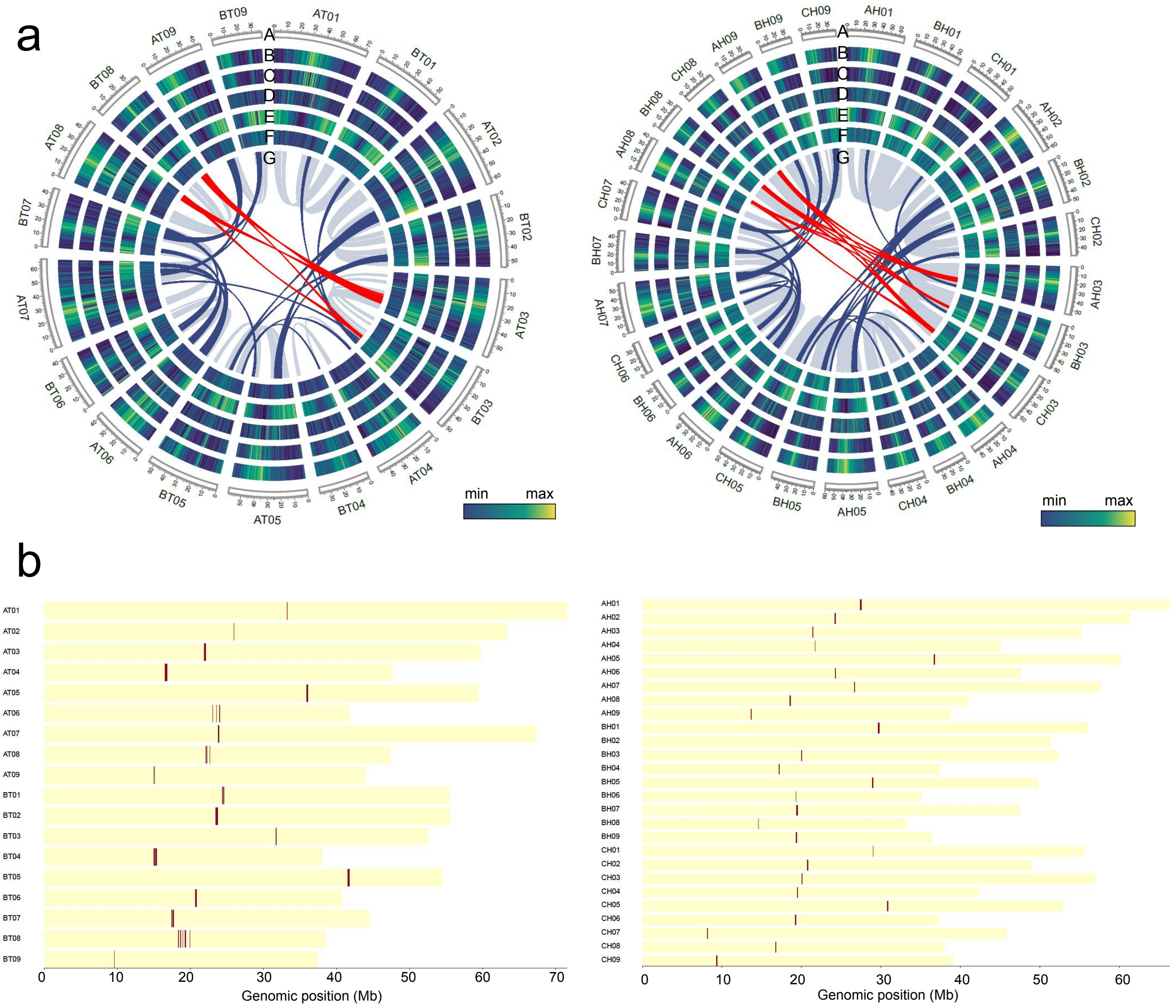

Supplementary Figure 8. Genomic features of *E. oryzicola* (ZJU2) and *E. crus-galli* (STB08) genomes.

a Circos plots of *E. oryzicola* (left) and *E. crus-galli* (right). The chromosomes are shown in track A. Track B-F show the densities of repeat elements (B), *Gypsy* (C), *Copia* (D), gene density (E) and GC content (F). Track G shows the syntenic blocks. Gray links show synteny among homologous subgenomes (only blocks larger than 100 syntenic gene pairs were shown), darkblue and red links show ancient synteny from a whole-genome duplication event shared by Poaceae.

b Centromeric regions identified using tandem repeats on each chromosome of *E. oryzicola* (left) and *E. crus-galli* genomes (right).

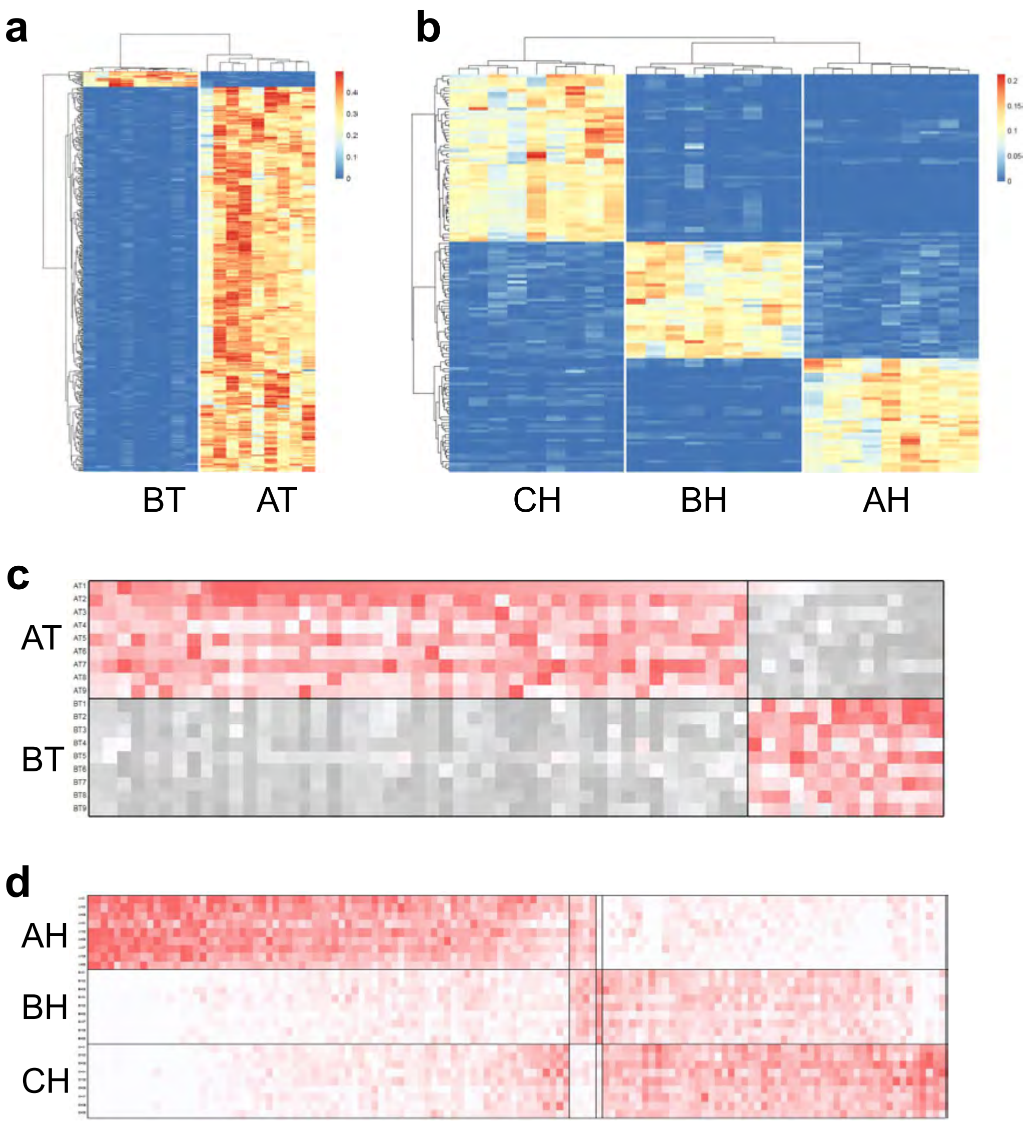

Supplementary Figure 9. Validation of subgenome distinguishing accuracy using *K*-mer and transposon elements analysis.
Clustering of counts of 13-mers that differentiate homeologous chromosomes enables the consistent partitioning of the genome into subgenomes in tetraploid *E. oryzicola* **(a)** and hexaploid *E. crus-galli.* **(b).** Transposon element abundance is used to confirm the accuracy of subgenome distinguishing in tetraploid *E. oryzicola* **(c)** and hexaploid *E. crus-galli* **(d)**. Subgenome-specific repeat elements would appear dominantly in the corresponding subgenome, as shown in red blocks for each chromosome. For example, in *E. oryzicola* genome, 61 transposon elements (including 2 LTR/Copia, 6 LTR/Gypsy and 53 other types) are found to distinguish subgenomes.

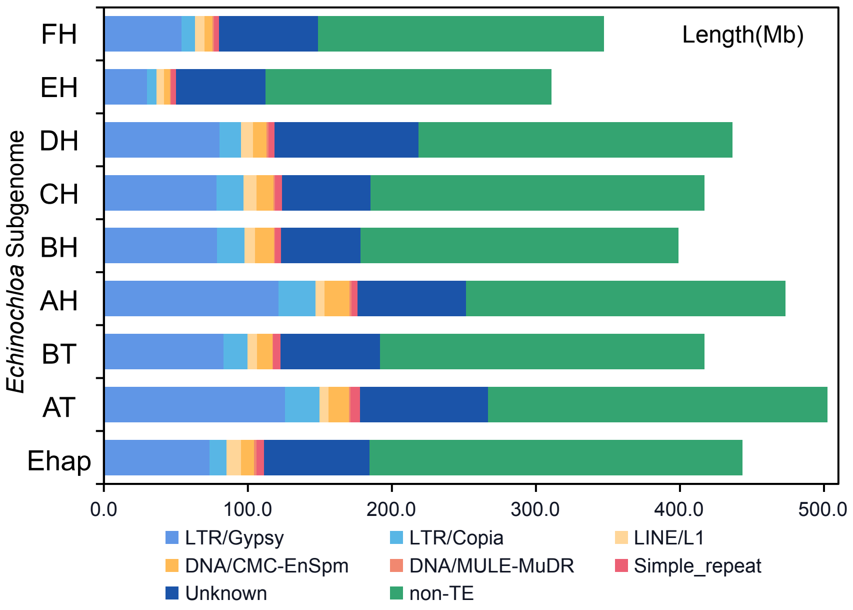

Supplementary Figure 10. Genomic compositions in each subgenome of *Echinochloa*. Ehap, diploid *E. haploclada*; AT and BT, two subgenomes in tetraploid *E. oryzicola*; AH, BH and CH, three subgenomes in hexaploid *E. crus-galli*; DH, EH and FH, three subgenomes in *E. colona*. Source data are provided as a Source Data file.

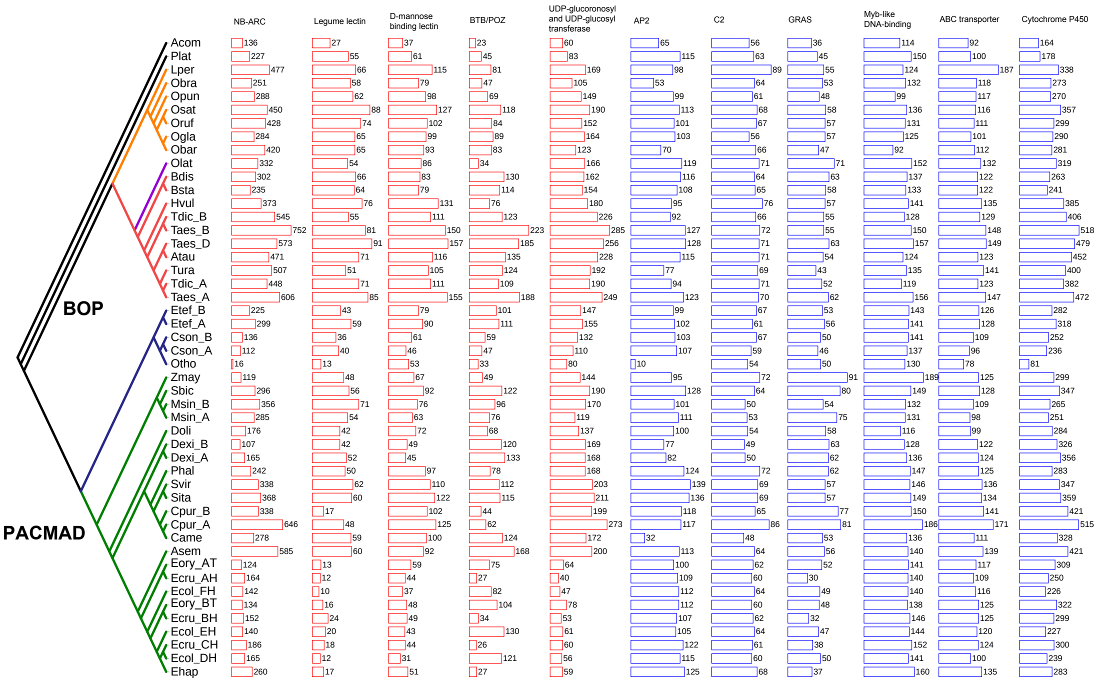

Supplementary Figure 11. Dynamics of gene family size in Poaceae.
Genomes of polyploidy species are split into diploid genomes (subgenomes). The phylogenetic tree is derived from 45 single-copy genes identified across 48 diploid genomes. It should be noticed that *Alloteropsis semialata* (Asem) and *Zea mays* (Zmay) have experienced recent whole-genome duplication or local duplication although they are diploid species in terms of the karyotype 2n=2×. (Sub)genome abbreviations refer to **Supplementary Data 1**.

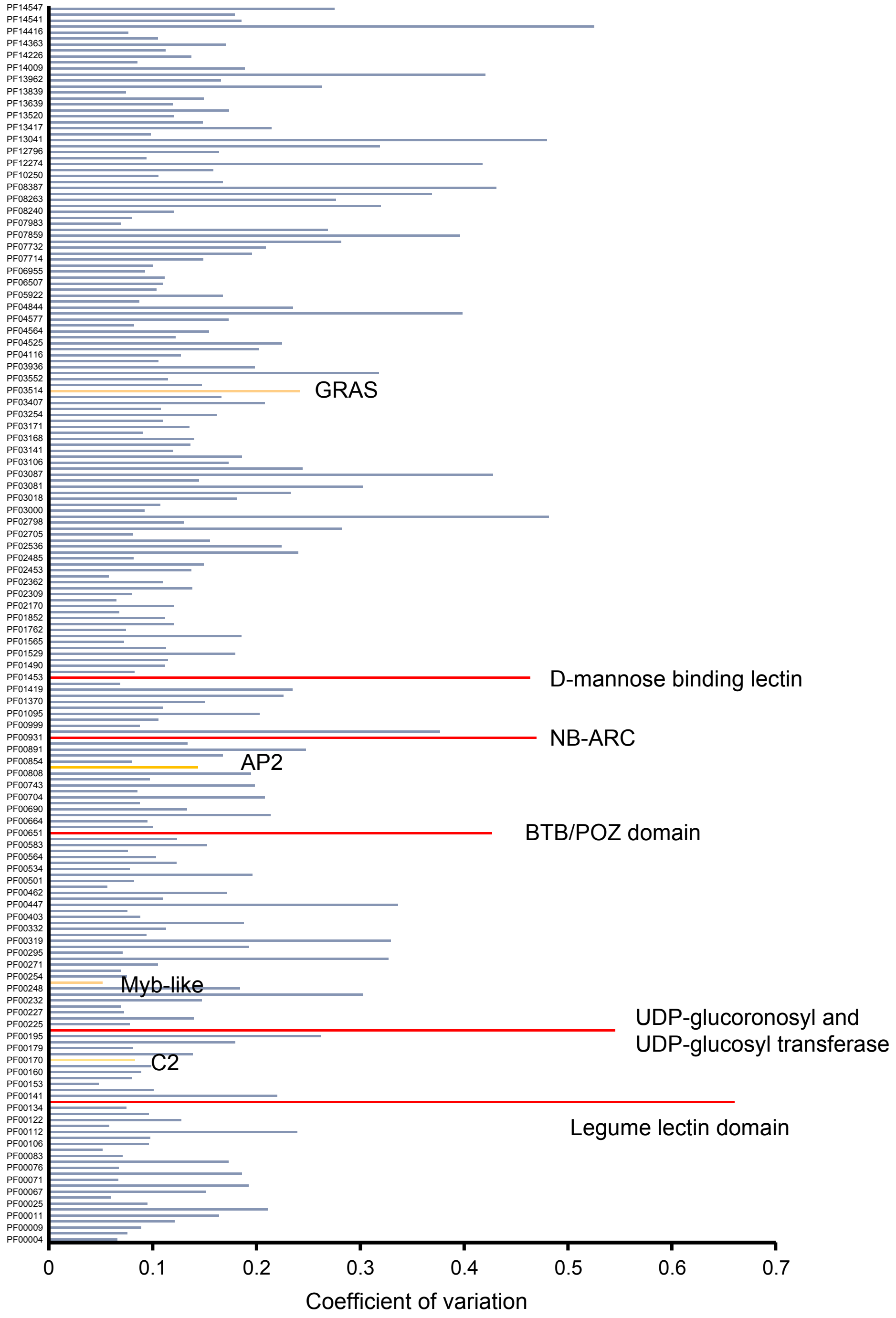

Supplementary Figure 12. Coefficient of variations (CVs) of gene family sizes across the Poaceae family. The gene families are determined by InterProScan. Gene families related to abiotic and biotic stress responses are marked in red and yellow, respectively.

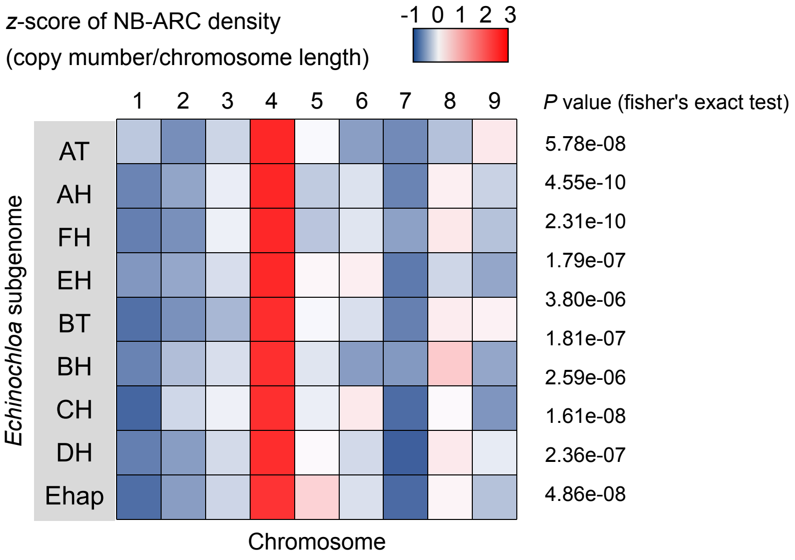

Supplementary Figure 13. NB-ARC genes are enriched on chromosomes 4 in *Echinochloa*.
The NB-ARC gene densities on 9 chromosomes from each subgenome are standardized using *z*-score. The *P* values are obtained using fisher's exact test. Source data are provided as a Source Data file.

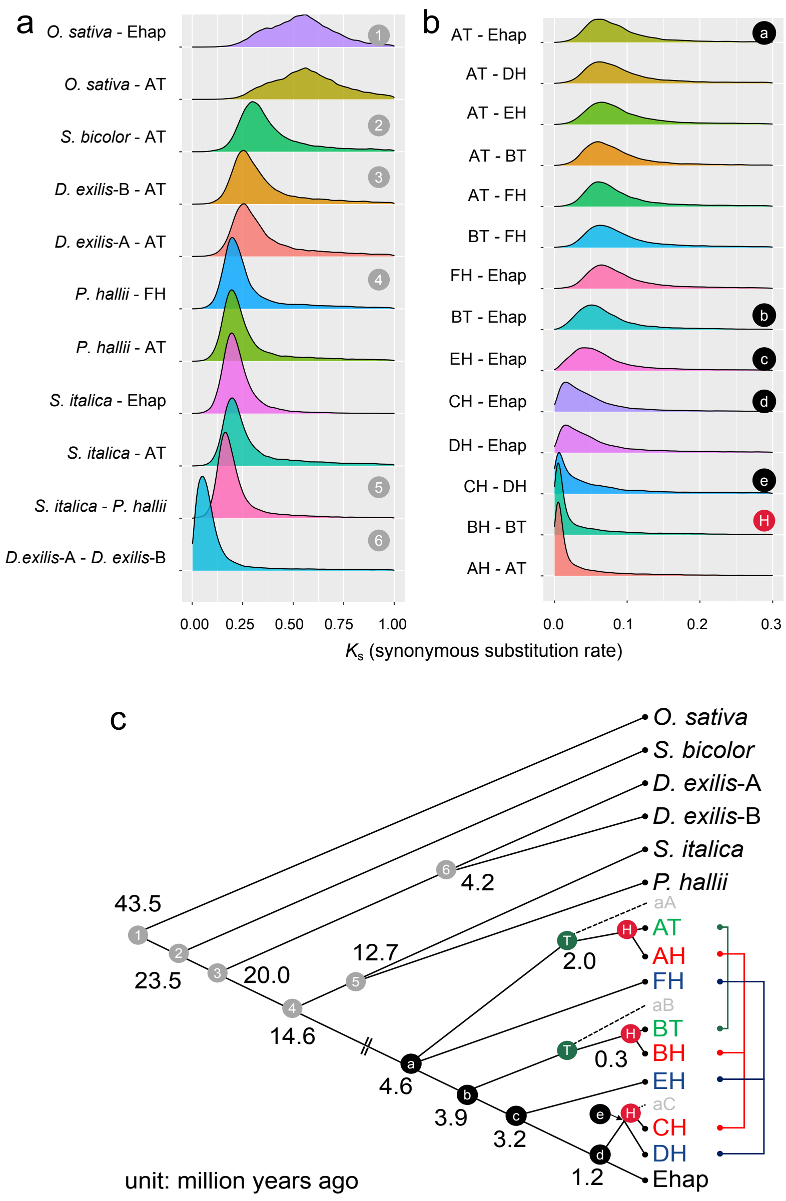

Supplementary Figure 14. The non-synonymous substitution rate K_s distributions based on syntenic gene pairs among *Echinochloa* and other genomes.

a K_s distributions between *Echinochloa* and other Poaceae (sub)genomes. **b** K_s distributions within *Echinochloa* subgenomes. **c** Phylogeny based on the K_s distributions among genomes. The numbers represent the divergence times estimated by K_s. "T" represents a tetraploidization event, and "H" represents a hexaploidization event.

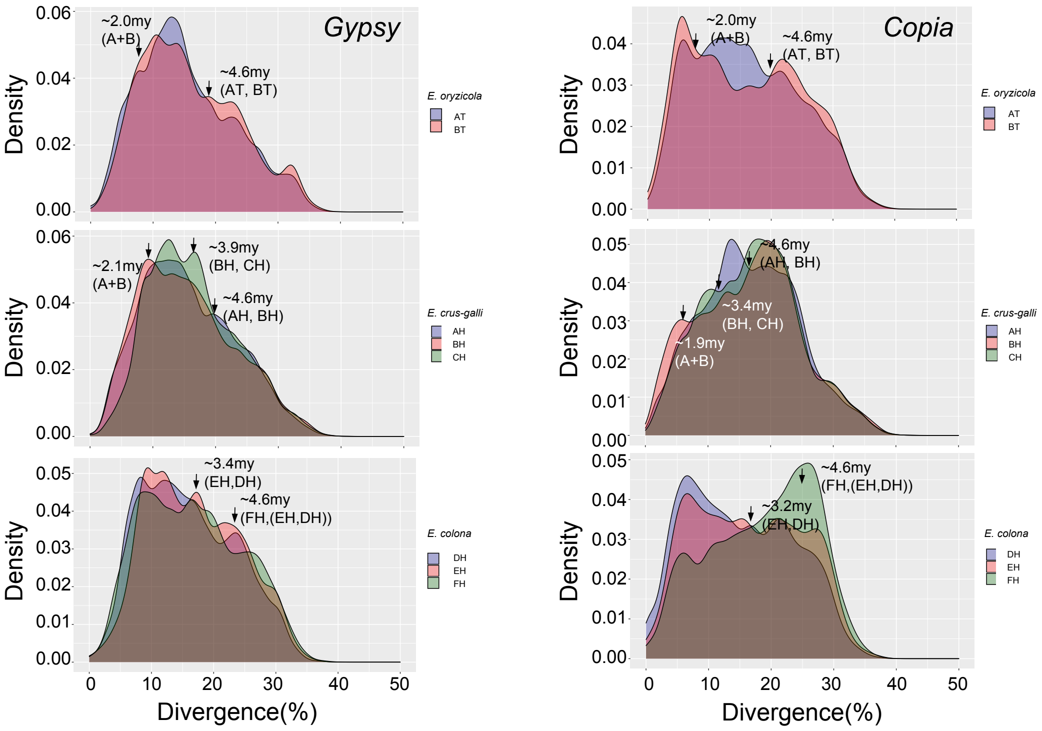

Supplementary Figure 15. Divergence time inferred from repeat elements divergence in *Echinochloa* polyploids.

Repeat elements in each subgenome are annotated by RepeatMask. Transposable elements divergence is assessed by PercDivs (Percentage of substitutions in the matching region compared to the consensus). my, million years.

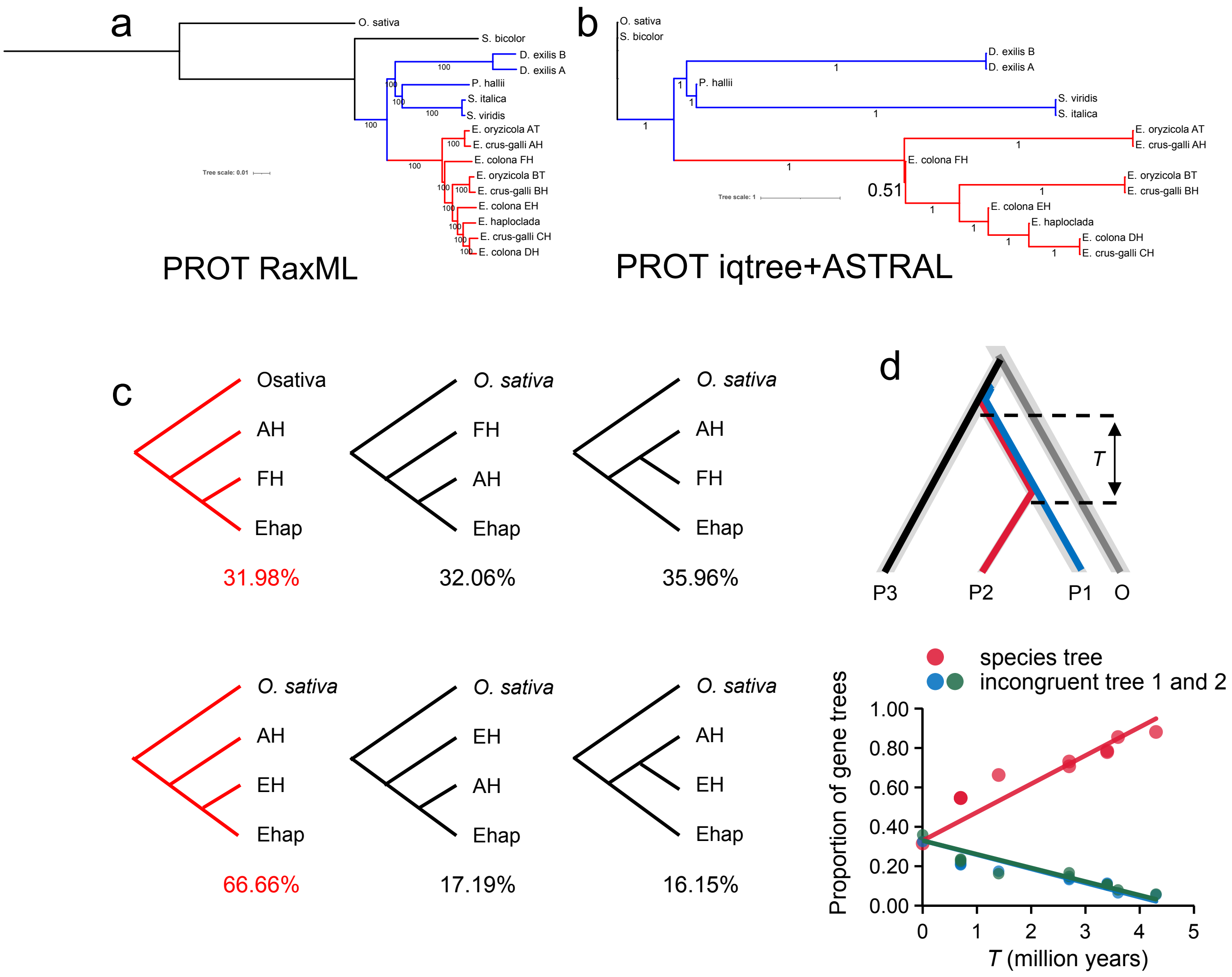

Supplementary Figure 16. ILS analysis in *Echinochloa*.

A phylogenic concatenation maximum-likelihood tree inferred from RaxML with the substitution model PROTGAMMA+JTT+F (**a**) and a phylogenetic tree based on single-copy gene trees by IQ-TREE (best substitution model tested by ModelFinder) and ASTRAL using 3557 single-copy genes are built (**b**). (**c**) The frequency of topologies around the first node of *Echinochloa* divergence (AH,FH, Ehap) and control (AH, EH, Ehap) with *O. sativa* as an outgroup. Species topologies are marked in red. (**d**) ILS footprints along with branch interval length. *T* represents the divergence interval in a triplet. With the interval increases, the incongruence caused by ILS decreases with 10.6% per million years. Source data are provided as a Source Data file.

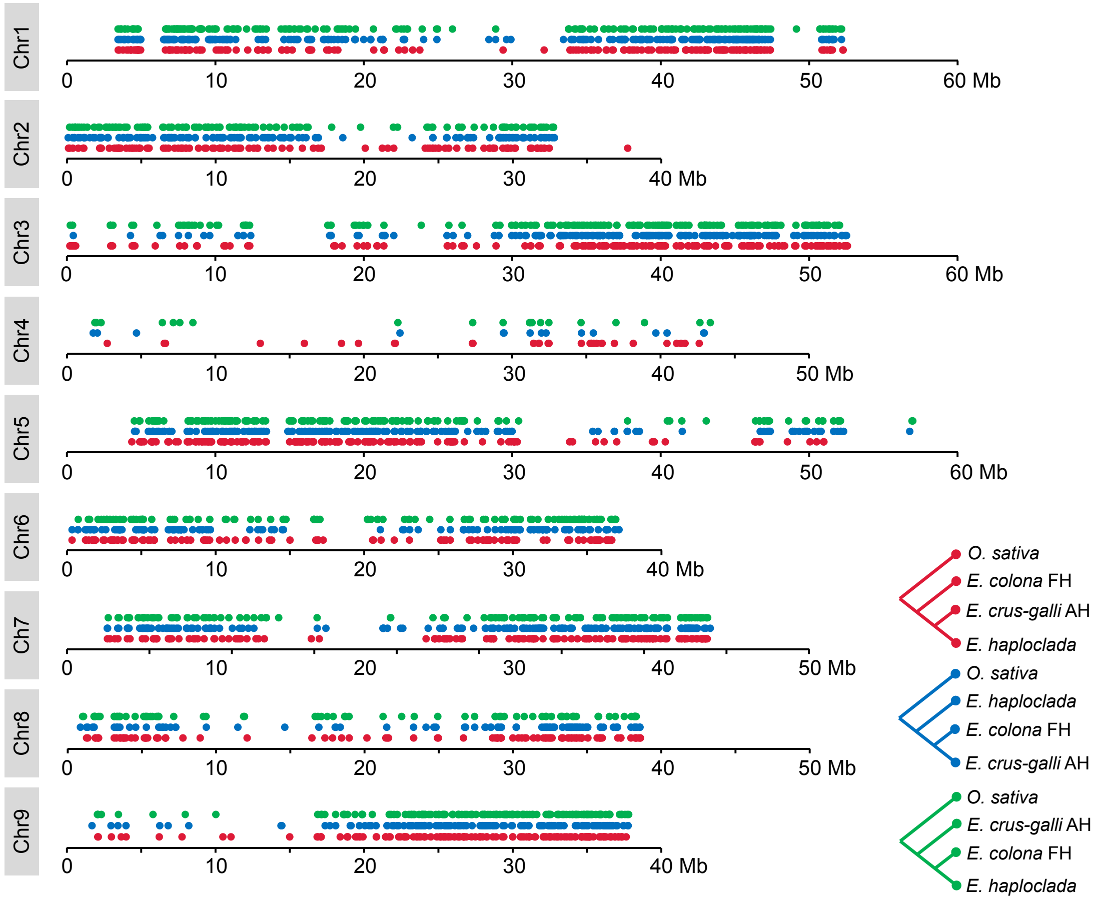

Supplementary Figure 17. Topology distribution of triplet (*O. sativa*, (*E. crus-galli* AH, *E. colona* FH, *E. haplocalda*)).

Dots along nine chromosomes with three different colors represent three topologies, respectively.

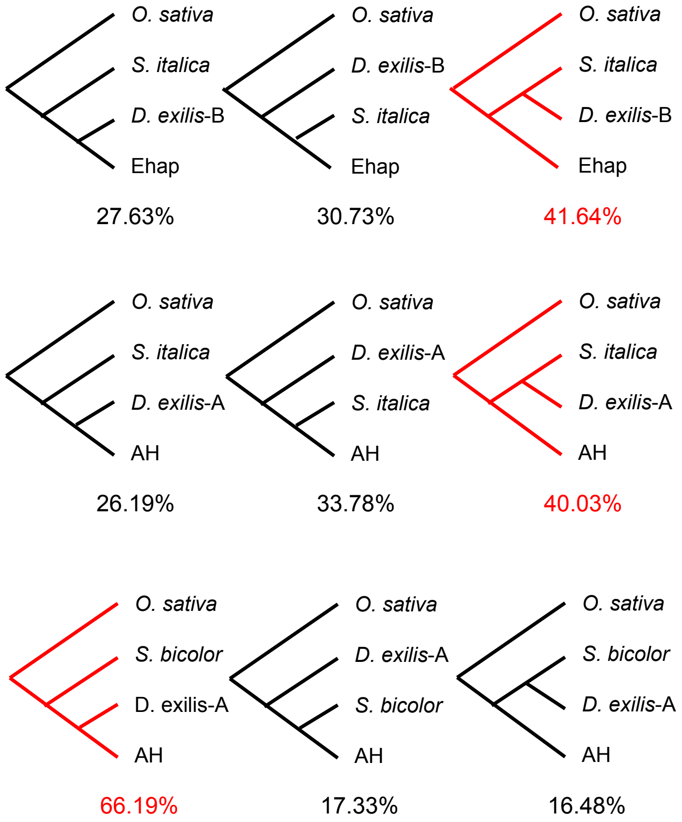

Supplementary Figure 18. The frequency of topologies among *Setaria* (*S. italica*), *Digitaria* (*D. exilis*-A and *D. exilis*-B), *Sorgum* (*S. bicolor*) and *Echinochloa* (Ehap and AH), with *Oryza* (*O. sativa*) as an outgroup.
Species topologies are marked in red.

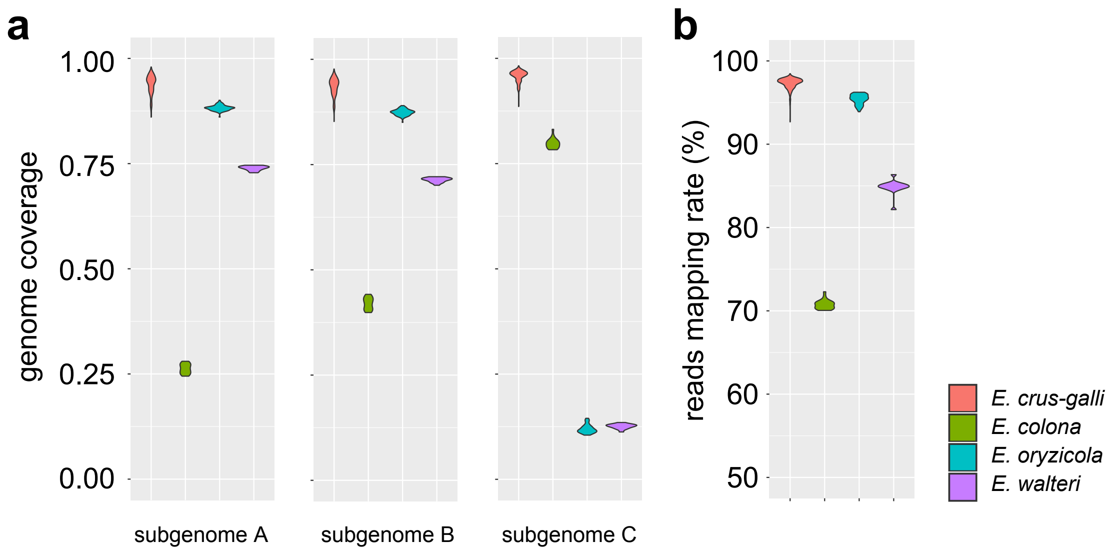

Supplementary Figure 19. Reads mapping for 737 re-sequenced *Echinochloa* accessions to *E. crus-galli* reference genome (STB08).

a Genome coverage of subgenome A, B and C for four species (*E. crus-galli*, *E. colona*, *E. oryzicola* and *E. walteri*). **b** Reads mapping rate for the four species.

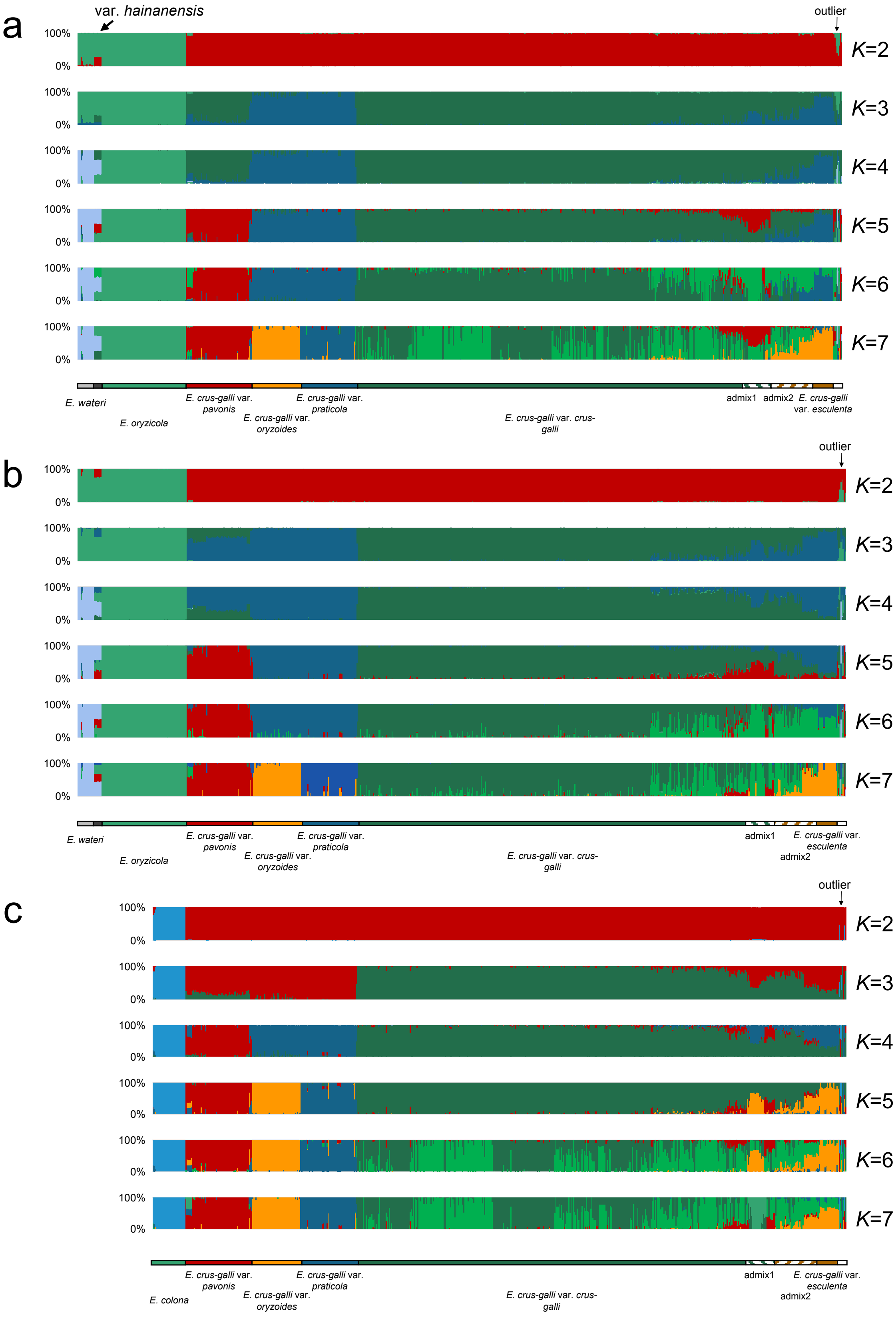

Supplementary Figure 20. Population structures from *K*=2 to *K*=7 based on whole-subgenome SNPs of subgenome A (a), B (b) and C (c), respectively.
Species or varieties names are indicated.

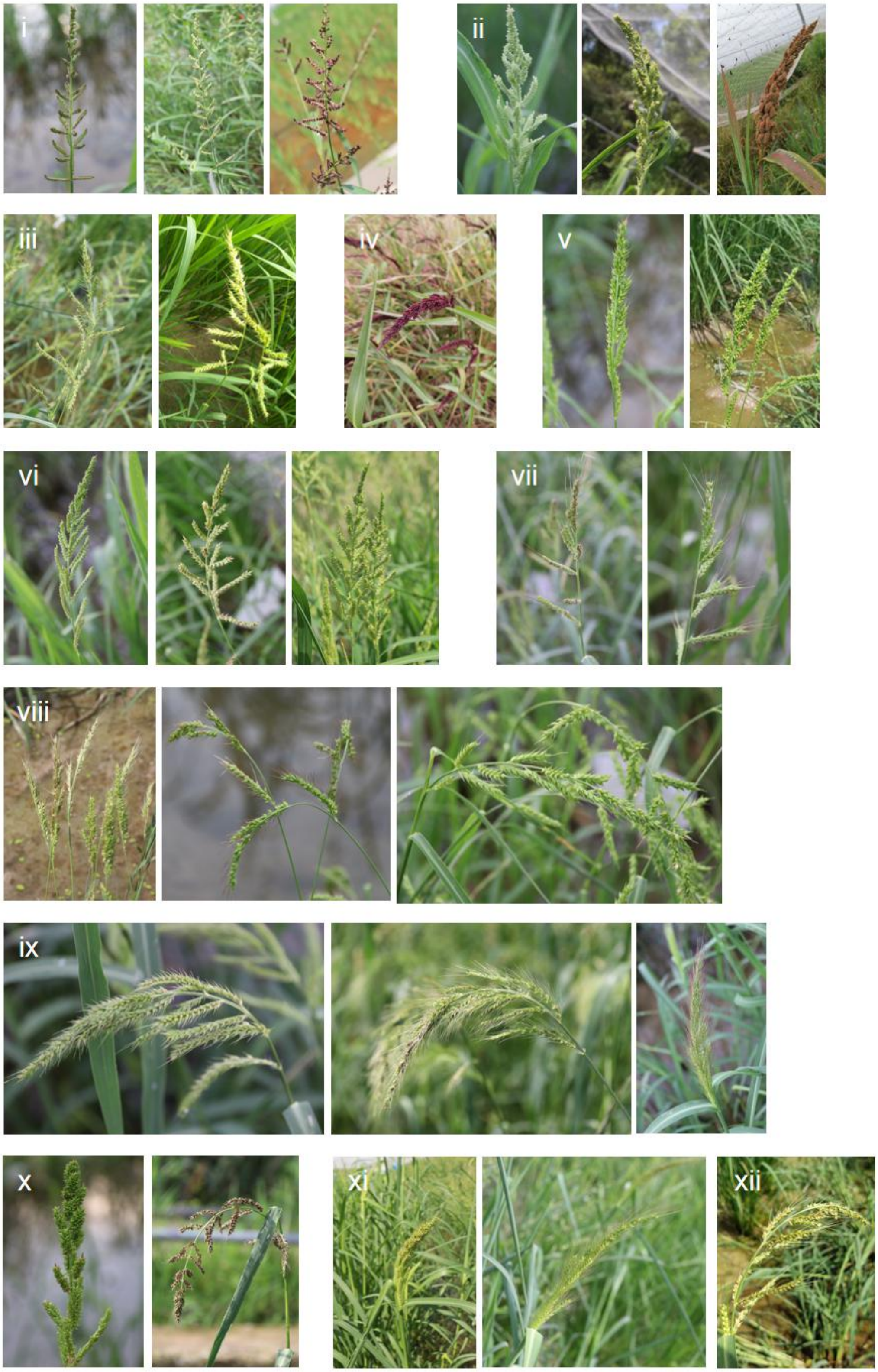

Supplementary Figure 21. Panicle phenotypes of *Echinochloa* species in this study.
i, *E. colona* var. *colona*. **ii**, *E. colona* var. frumentacea. **iii**, *E. walteri*. **iv**, *E. oryzicola* var. *hainanensis*. **v**, *E. oryzicola* var. *oryzicola*. **vi**, *E. crus-galli* var. *crus-galli*. **vii**, *E. crus-galli* var. *praticola*. **viii**, *E. crus-galli* var. *oryzoides*. **ix**, *E. crus-galli* var. *crus-pavonis*. **x**, *E. crus-galli* var. *esculenta*. **xi**, *E. crus-galli* (admix1). **xii**, *E. crus-galli* (admix2).

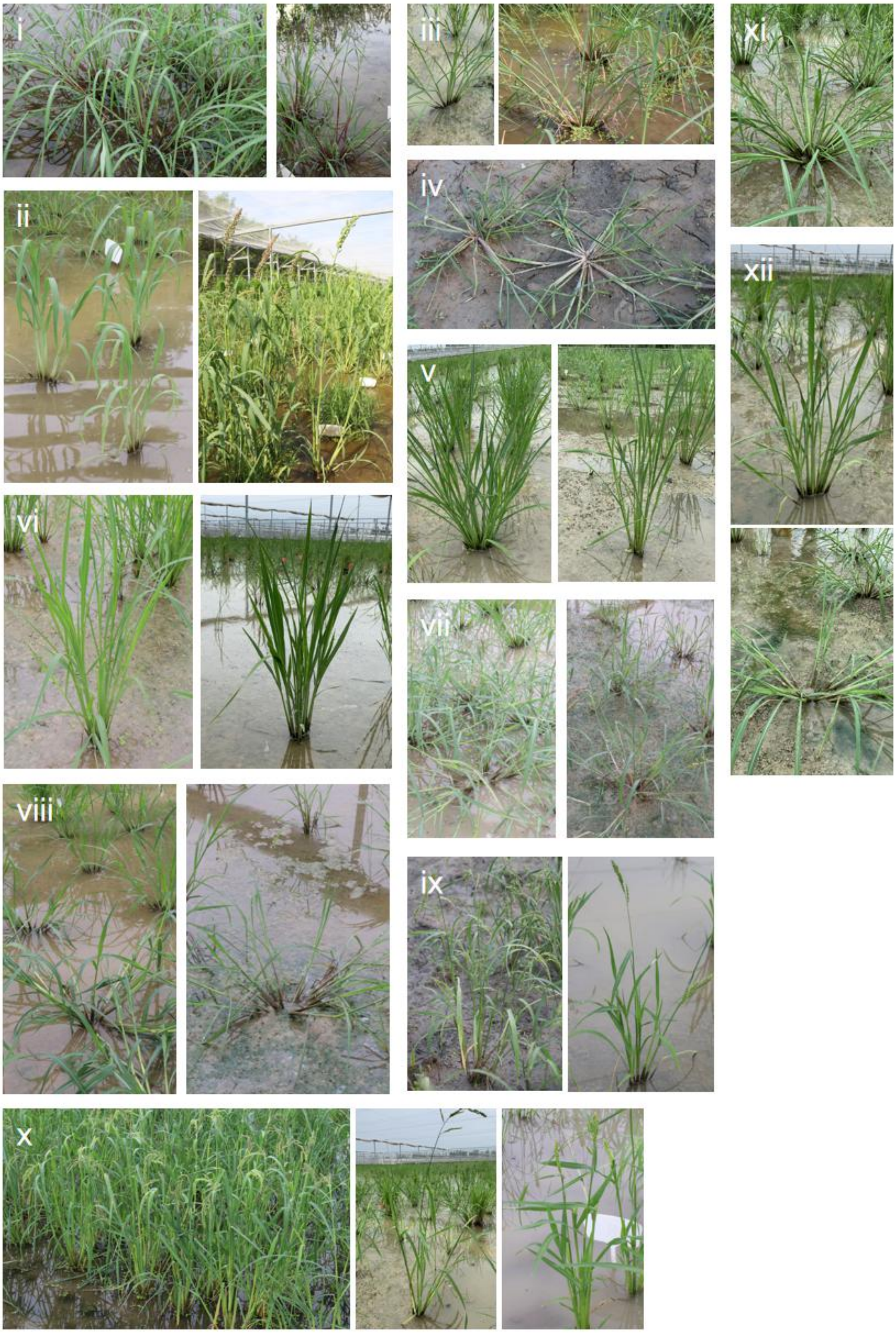

Supplementary Figure 22. Plant architectures of *Echinochloa* species in this study.
i, *E. colona* var. *colona*. **ii**, *E. colona* var. frumentacea. **iii**, *E. walteri*. **iv**, *E. oryzicola* var. *hainanensis*. **v**, *E. oryzicola* var. *oryzicola*. **vi**, *E. crus-galli* var. *crus-galli*. **vii**, *E. crus-galli* var. *praticola*. **viii**, *E. crus-galli* var. *crus-pavonis*. **ix**, *E. crus-galli* var. *oryzoides*. **x**, *E. crus-galli* var. *esculenta*. **xi**, *E. crus-galli* (admix2). **xii**, *E. crus-galli* (admix1).

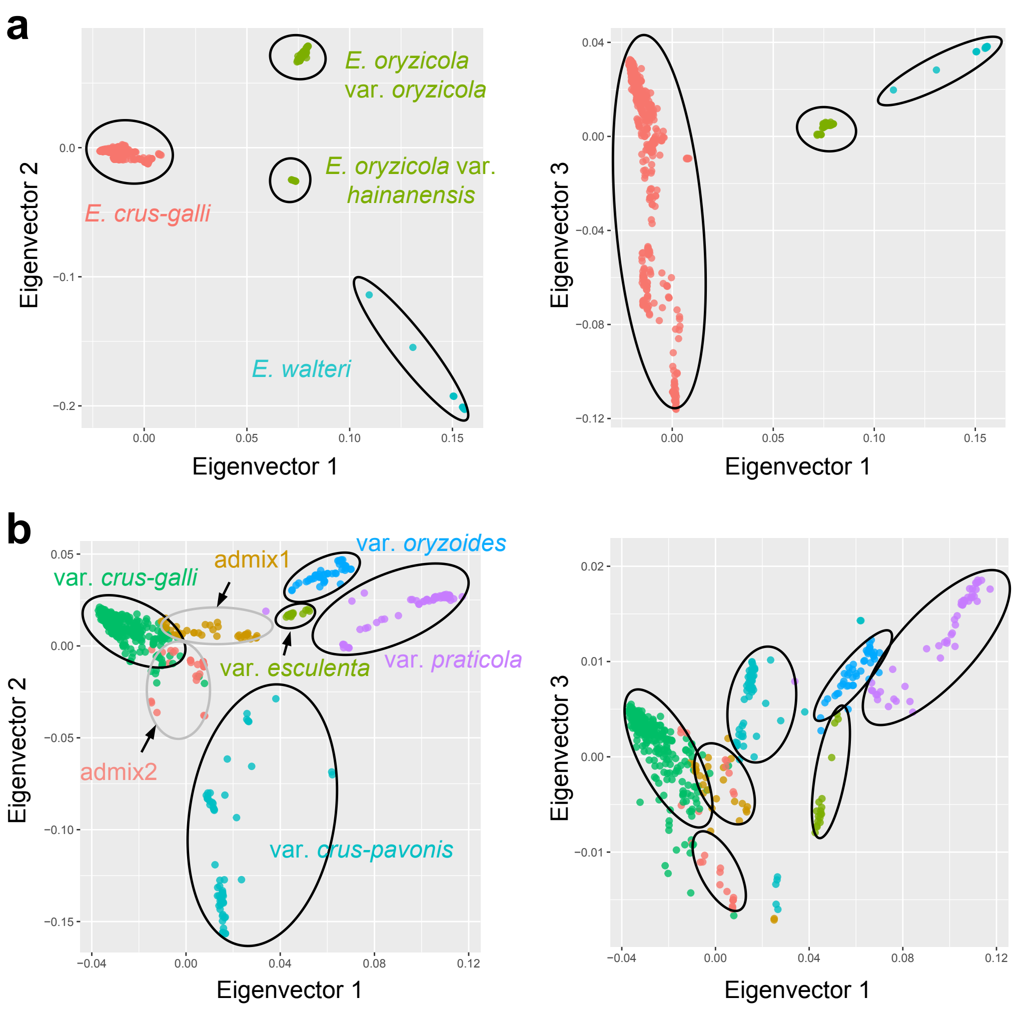

Supplementary Figure 23. PCA analysis of all *Echinochloa* individuals (a) and *E. crus-galli* individuals (b) using whole-genome pruned SNPs.

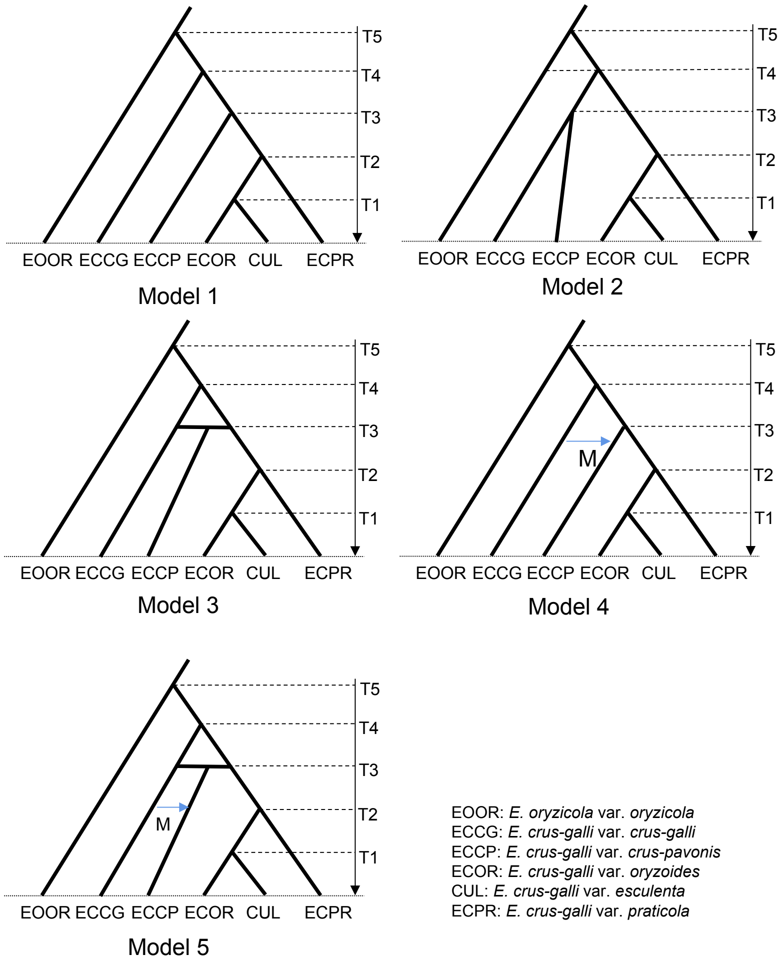

Supplementary Figure 24. Five scenarios for divergence of five *E. crus-galli* varieties with *E. oryzicola* var. *oryzicola* as an outgroup.

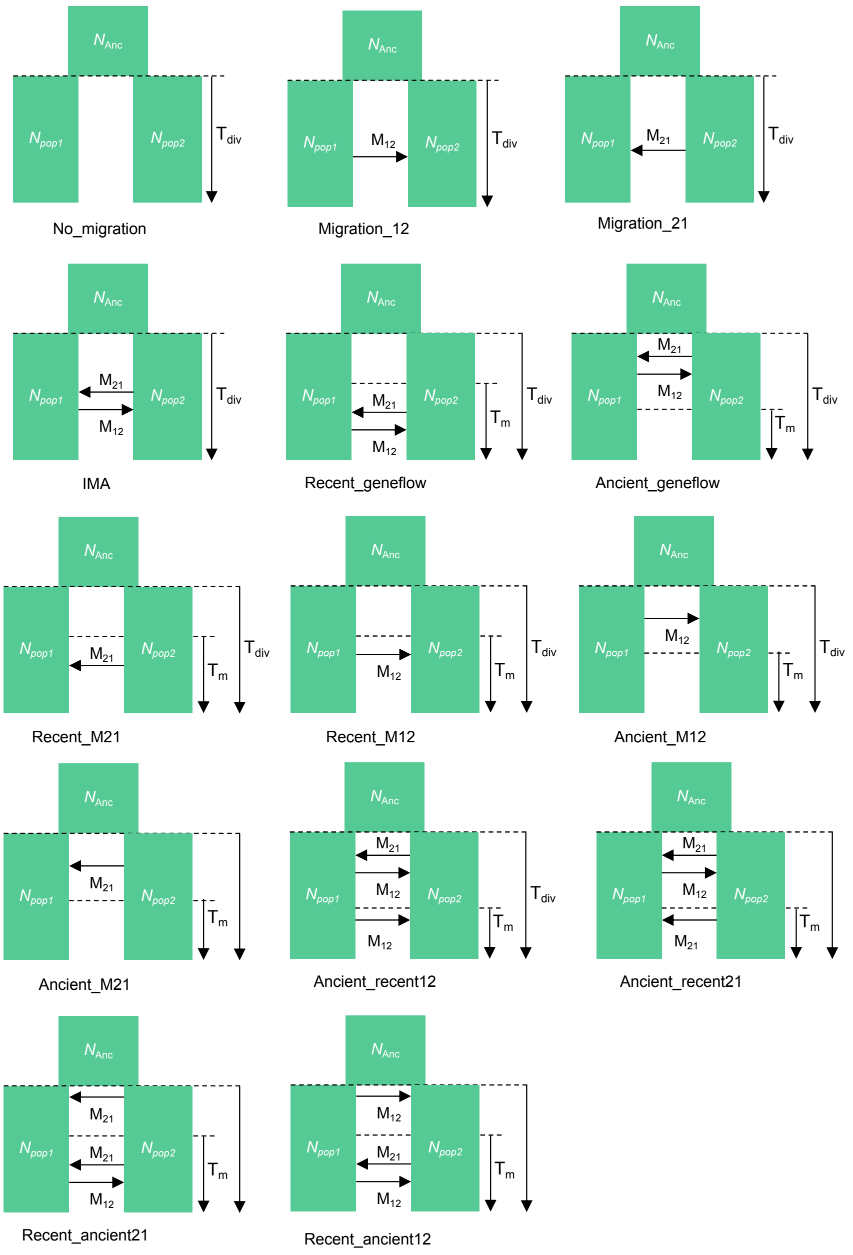

Supplementary Figure 25. Fourteen scenarios to test the existence and directions of gene flow between groups.

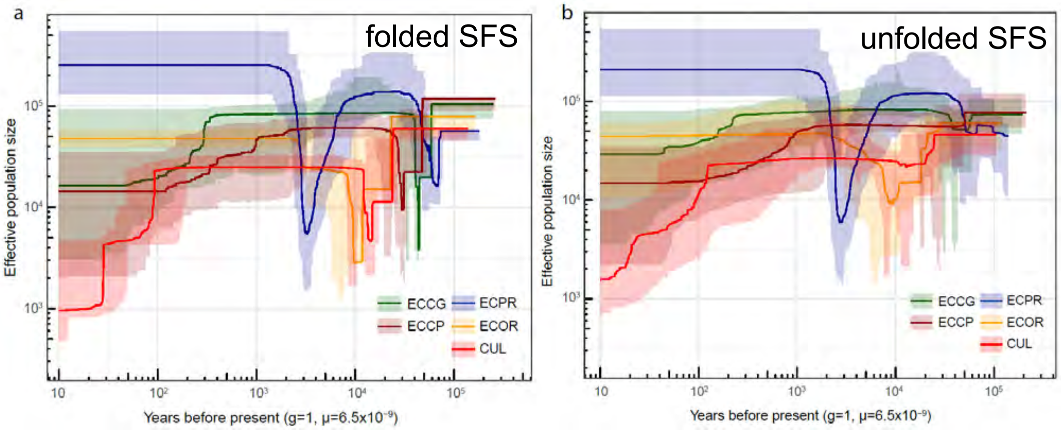

Supplementary Figure 26. Recent demographic history of five *E. crus-galli* varieties inferred by Stairway Plot using folded SFS (a) and unfolded SFS (b).

The line indicates the median of effective population size N_e estimation based on 200 inferences, and the error band indicates 95% confidence interval for each population. ECCG, *E. crus-galli* var. *crus-galli.* ECCP, *E. crus-galli* var. *crus-pavonis.* ECOR, *E. crus-galli* var. *oryzoides.* CUL, *E. crus-galli* var. *esculenta.* ECPR, *E. crus-galli* var. *praticola.*

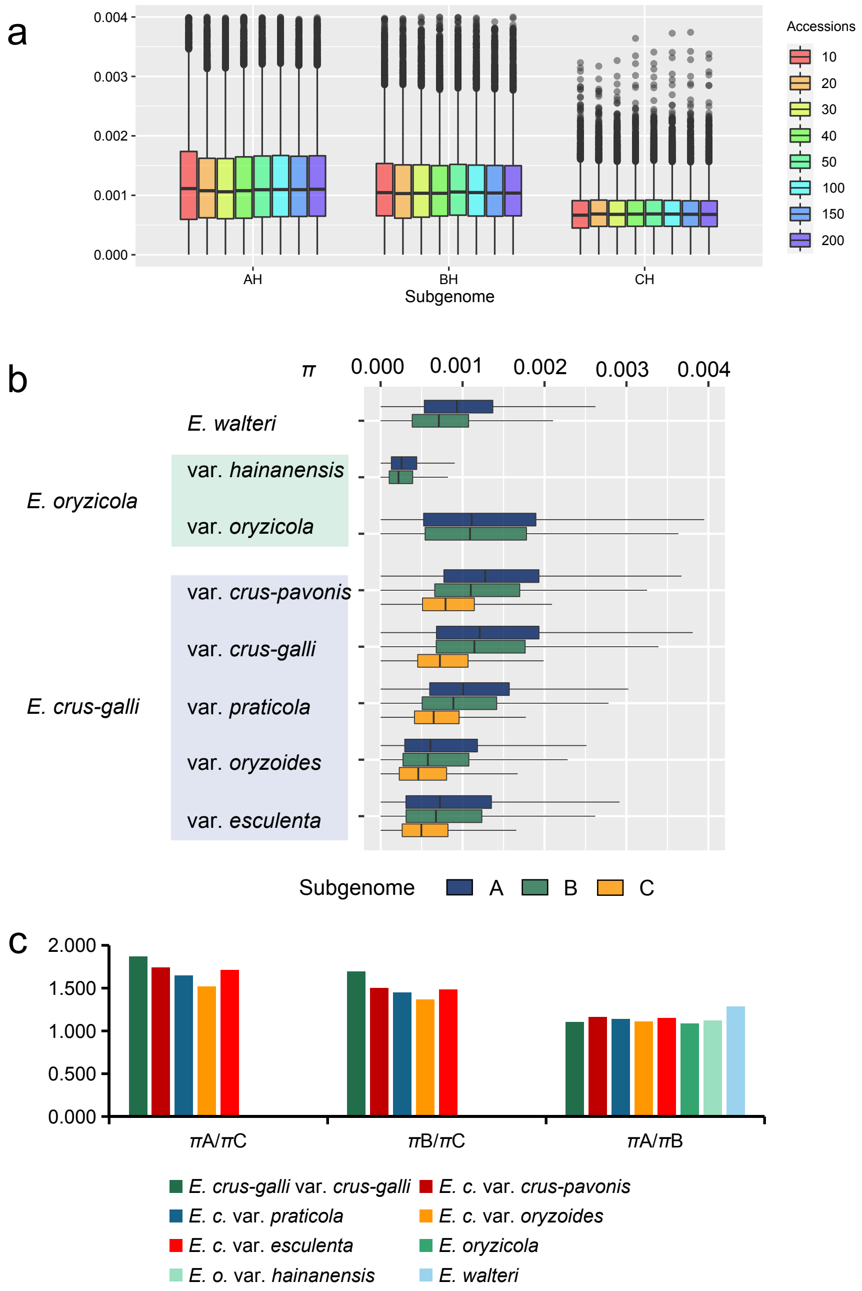

Supplementary Figure 27. Nucleotide diversity in *Echinochloa*.

a The effects of sampling size on nucleotide diversity (π) estimation. Populations with sampling size of 10, 20, 30, 40, 50, 100, 150 and 200 were randomly selected from *E.crus-galli* var. *crus-galli*, respectively. The nucleotide diversity was estimated in each subgenome (100-kb sliding windows with a 50-kb step: n = 9396 AH, n = 7925 BH, n = 8305 CH). In the box plots, the horizontal line shows the median value, and the whiskers show the 25% and 75% quartile values. **b** Nucleotide diversity (π) of *Echinochloa* species or varieties at subgenome level. 20-kb sliding windows: n = 22860, n = 19221 for B, n = 20238 for C. In the box plots, the vertical line shows the median value, and the whiskers show the 25% and 75% quartile values. **c** Comparisons among subgenomes of nucleotide diversity (π) in eight *Echinochloa* species or varieties.

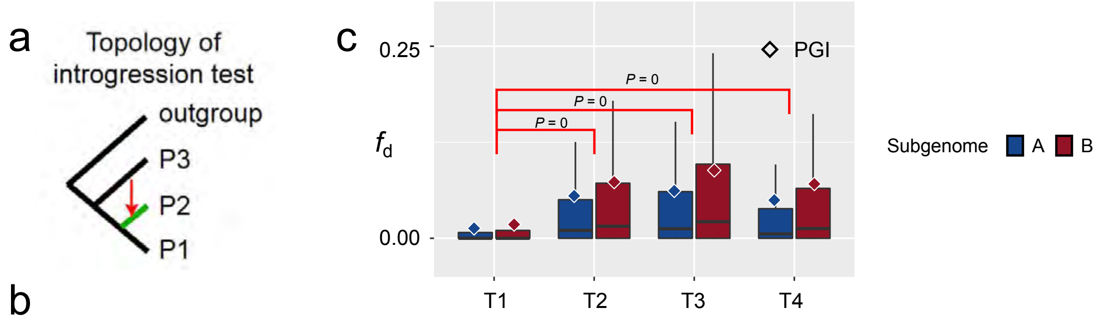

b

Topology	T1	T2	T3	T4
outgroup	*E. walteri*	*E. walteri*	*E. walteri*	*E. o.* var. *hainanensis*
P3	*E. o.* var. *oryzicola*	*E. o.* var. *oryzicola*	*E. o.* var. *oryzicola*	*E. o.* var. *oryzicola*
P2	*E. c.* var. *praticola*	*E. c.* var. *crus-galli*	*E. c.* var. *crus-galli*	*E. c.* var. *crus-galli*
P1	*E. c.* var. *oryzoides*	*E. c.* var. *oryzoides*	*E. c.* var. *praticola*	*E. c.* var. *oryzoides*

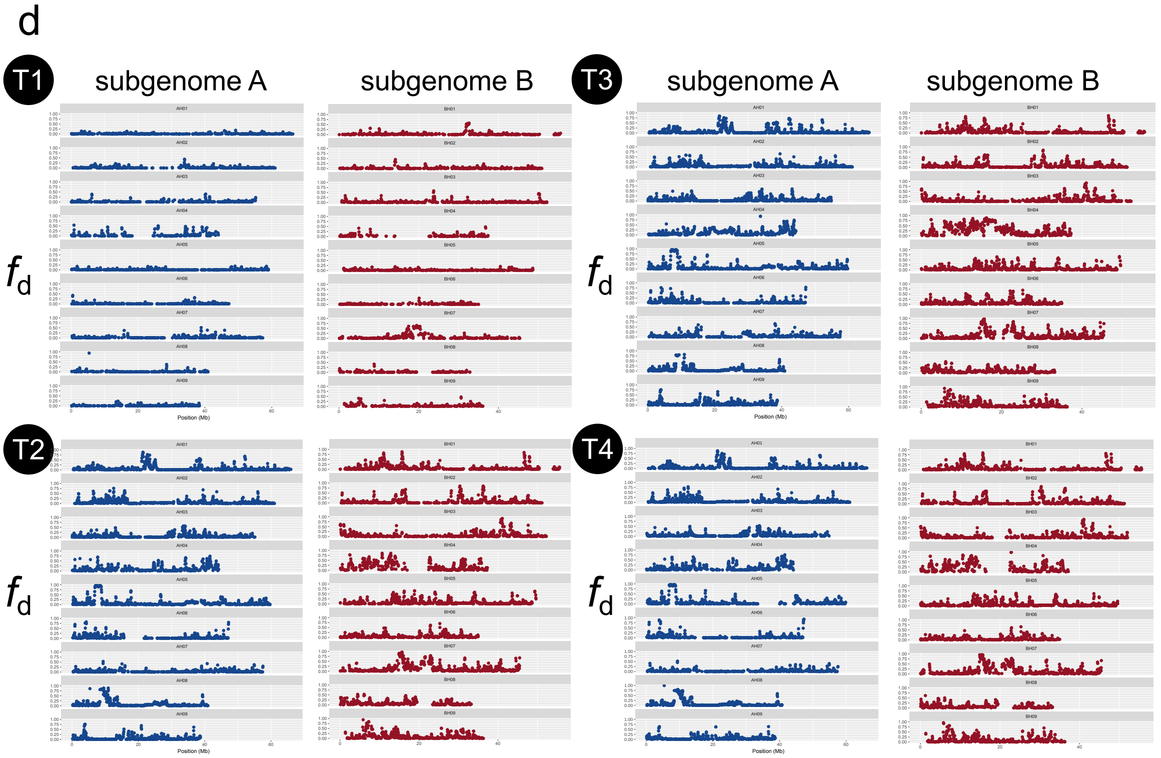

Supplementary Figure 28. Introgression analysis using ABBA-BABA test.
a Brief illustration of ABBA-BABA topology. Red arrow indicates the introgression direction from P3 to P2. **b** Four topologies (T1-T4) are set to test. **c** Genome-wide overall f_d and PGI (proportion of genomic introgression) in topology T1-T4. Numbers of 100-kb windows with a step of 50kb: n = 8543 for subgenome A and n = 7197 for subgenome B. In the box plots, the horizontal line shows the median value, and the whiskers show the 25% and 75% quartile values of f_d. P values were calculated using the two-tailed t-test. **d** f_d distribution along chromosomes in four topologies (T1-T4).

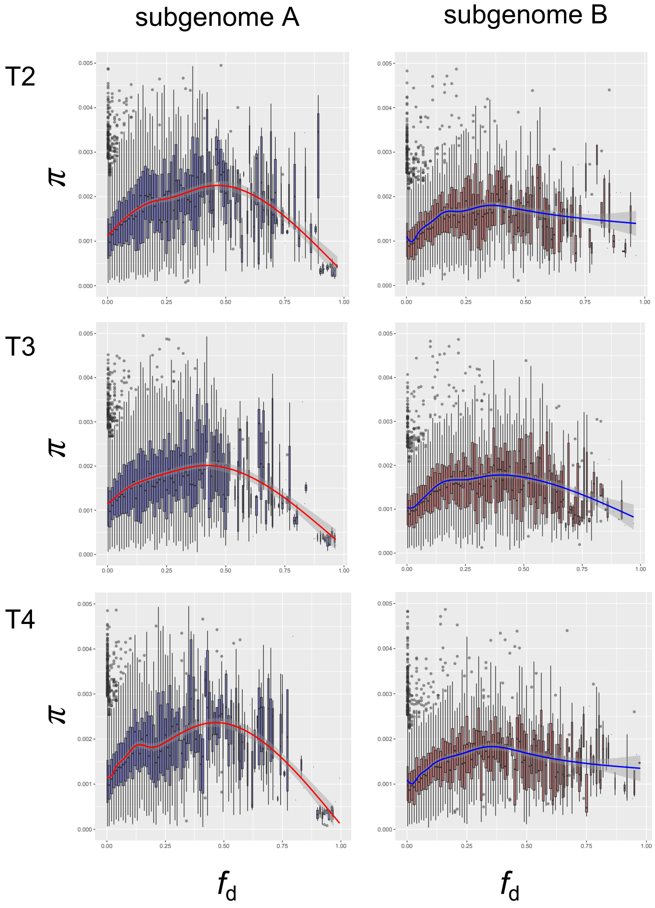

Supplementary Figure 29. Relationship between nucleotide diversity (π) and introgression (f_d). Sliding window size of f_d is 0.01. In the box plots of f_d windows, the horizontal line shows the median value, and the whiskers show the 25% and 75% quartile values of diversity π. The red and blue lines are the fitting curves between f_d and π and gray bands are 95% confidence intervals. See details of topology T2-T4 in **Supplementary Fig. 28**.

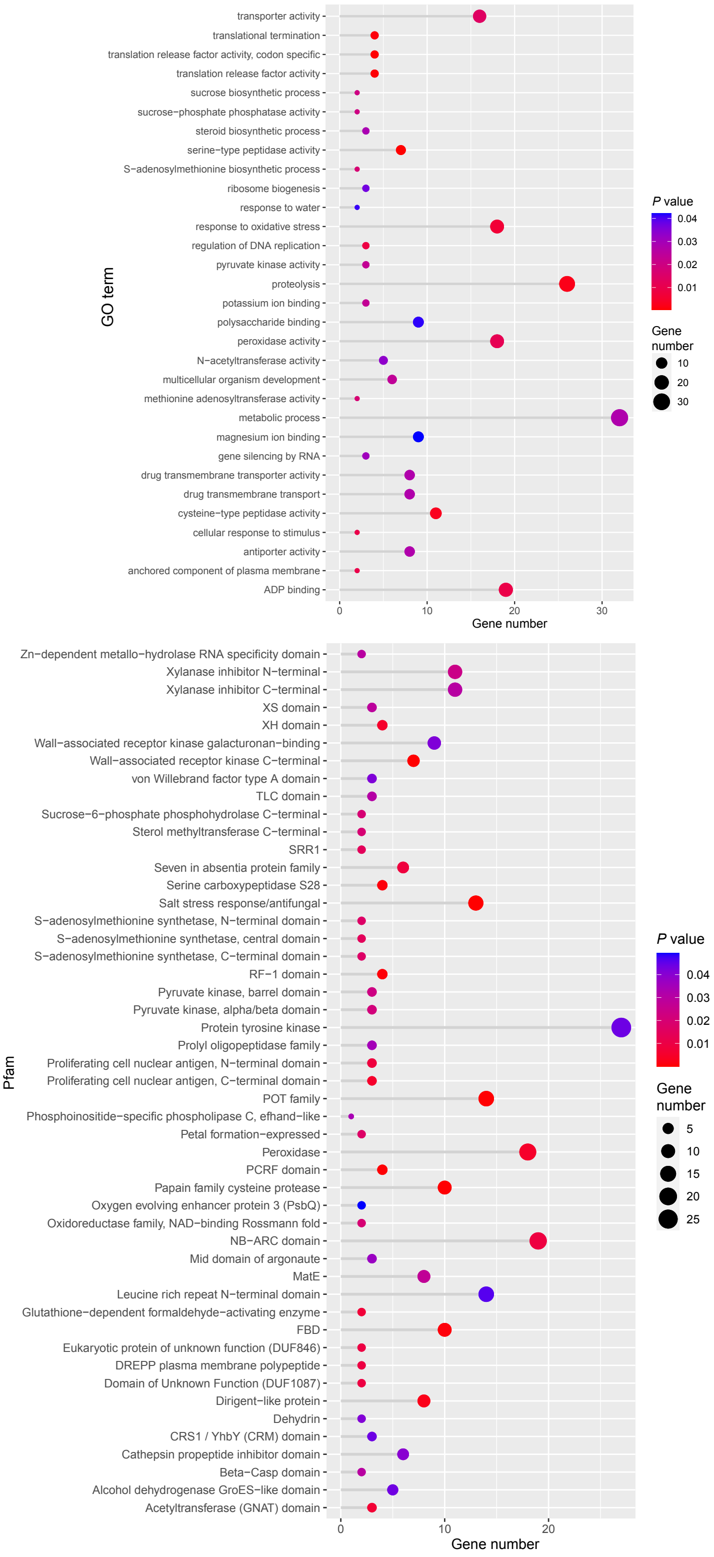

Supplementary Figure 30. Function (gene ontology and Pfam domain) enrichments of introgressed segments from *E. oryzicola* var. *oryzicola* to *E. crus-galli* var. *crus-galli* with f_d > 0.5.

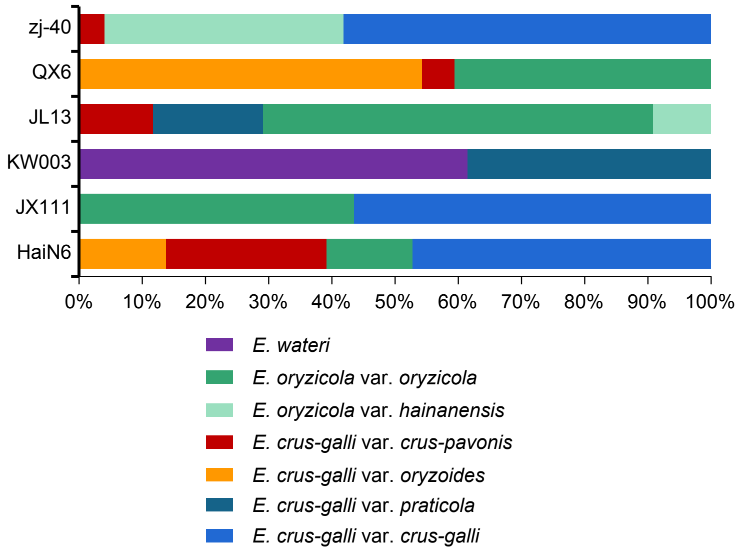

Supplementary Figure 31. Genomic compositions of outlier *Echinochloa* accessions (inferred by population structure using SNPs from subgenomes A and B when *K* = 7). Different colors represent genetic components of seven *Echinochloa* species or varieties.

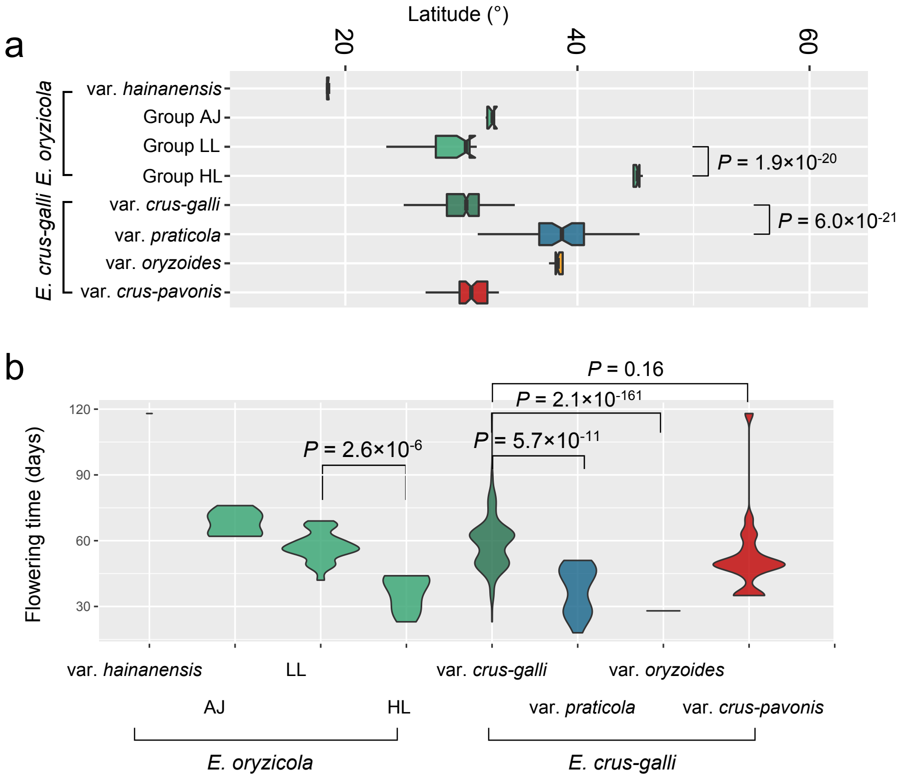

Supplementary Figure 32. Latitude distribution (a) and flowering time in 2017 (b) of *Echinochloa* varieties or groups.

Group size: *n* = 7 for var. *hainanensis*, *n* = 7 for AJ, *n* = 34 for LL, *n* = 37 for HL, *n* = 356 for var. *crus-galli*, *n* = 51 for var. *praticola*, n = 44 for var. *oryzoides*, and *n* = 61 for var. *crus-pavonis*. In the box plots, the vertical line shows the median value, and the whiskers show the 25% and 75% quartile values of latitudes from each group. *P* values were obtained by the two-tailed *t*-test.

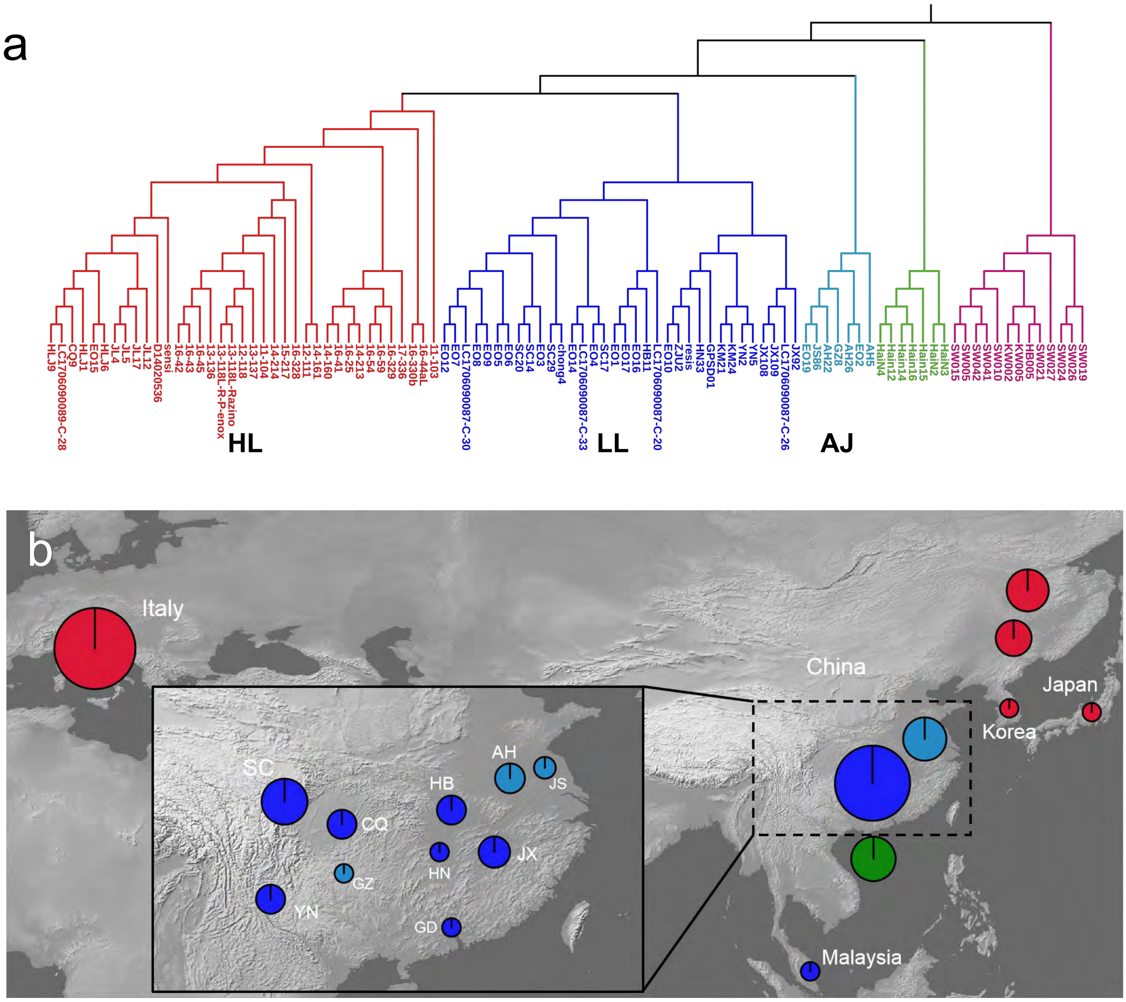

Supplementary Figure 33. A maximum-likelihood phylogenetic tree and geographical distribution of *E. oryzicola* individuals.

a A ML phylogenetic tree of *E. oryzicola*, inferring from whole-genome 7,312,107 SNPs, with *E. walteri* (purple) as an outgroup. Green, sky-blue, blue and red lines represent var. *hainanensis*, group AJ (Anhui and Jiangsu), group LL (low latitude) and group HL (high latitude), respectively. **b** Geographical distributions of *E. oryzicola* groups.

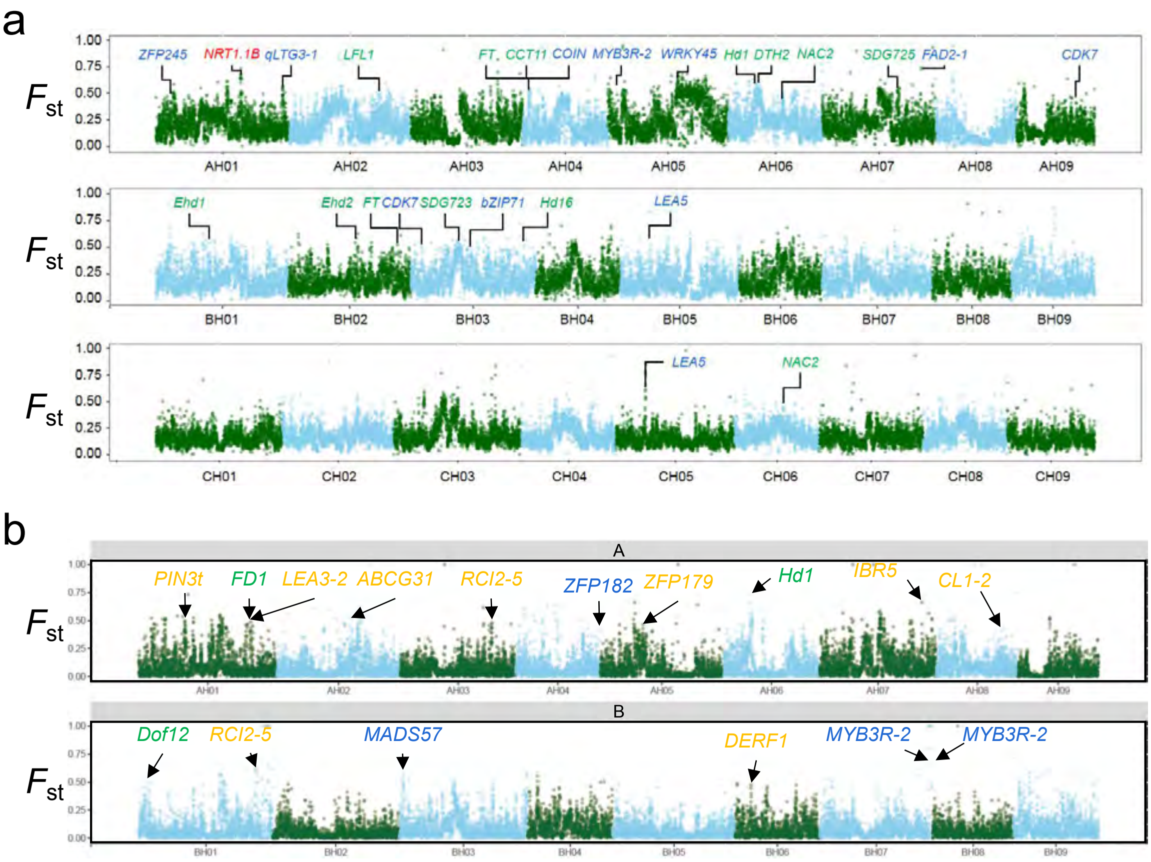

Supplementary Figure 34. Genomic differentiation analysis.

a F_{st} between *E. crus-galli* var. *crus-galli* and var. *praticola*. **b** F_{st} between *E. oryzicola* group LL and HL. Genes related to flowering time, cold response, and drought response are marked in green, blue and yellow, respectively.

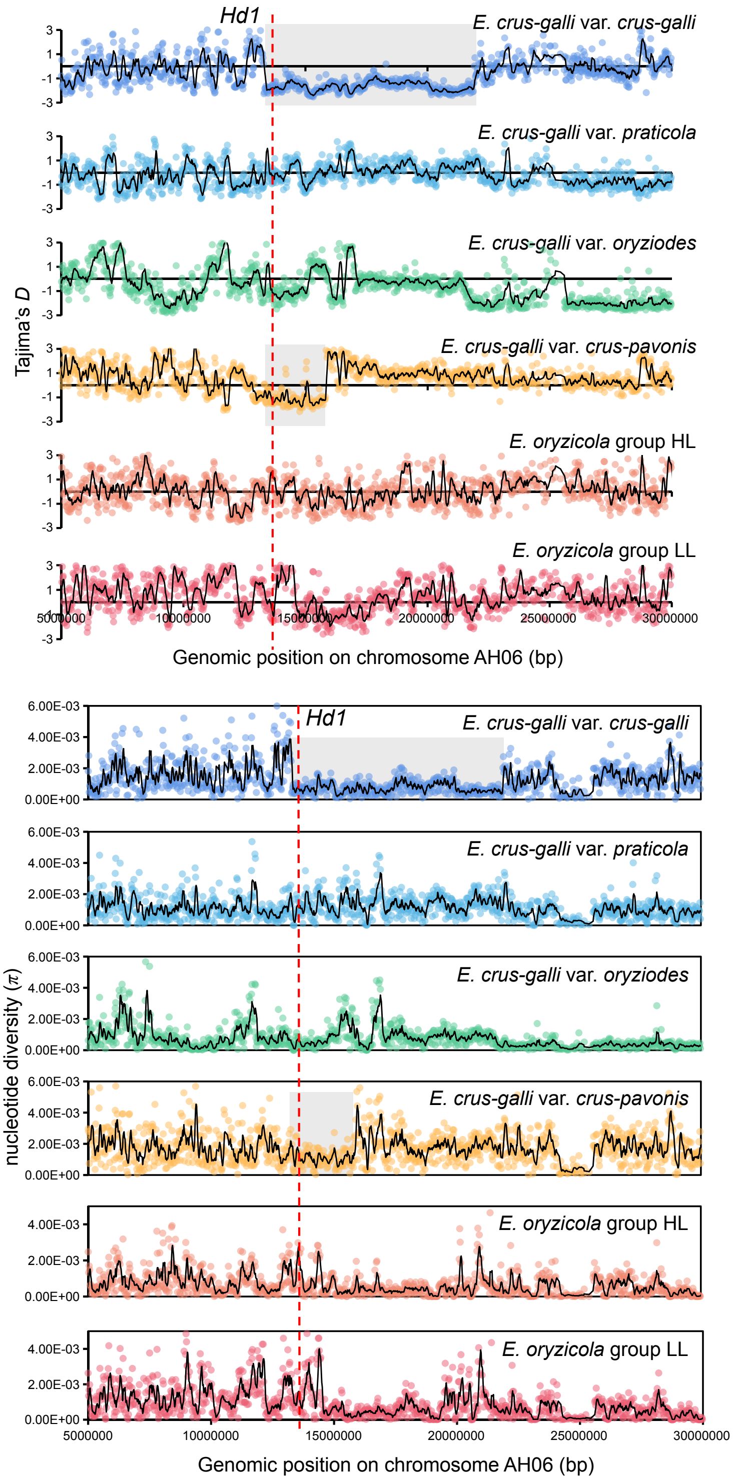

Supplementary Figure 35. Tajima's *D* values and nucleotide diversity (*π*) of *E. crus-galli* varieties and *E. oryzicola* groups from 5 Mb to 30 Mb on chromosome AH06.
Sliding window size is set as 20 kb.

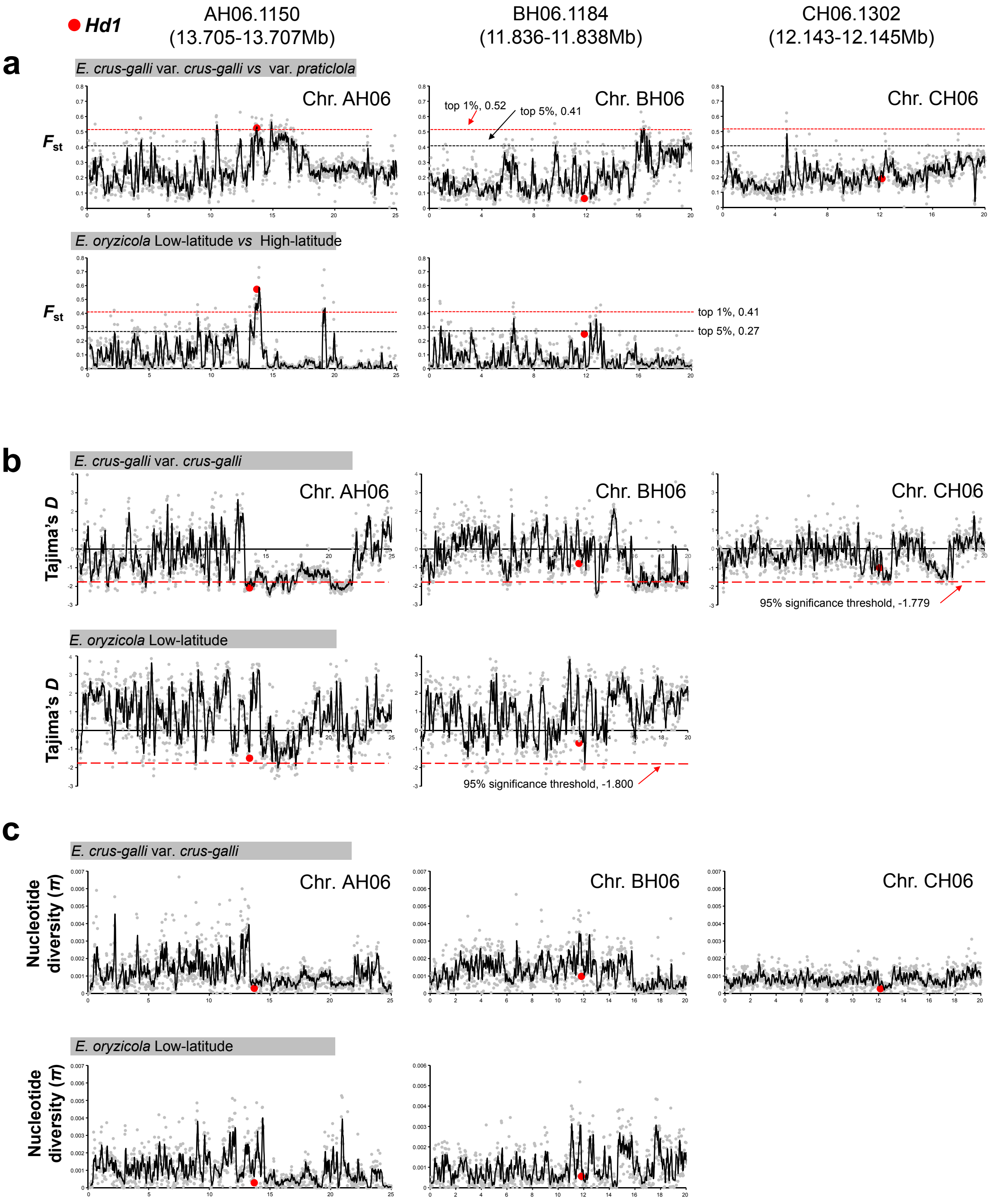

Supplementary Figure 36. Preferential differentiation and selection on *Hd1* related to flowering time in *E. crus-galli* and *E. oryzicola*.

a Genomic differentiation scan in 20-kb sliding windows around three orthologs of *Hd1* (AH06.1150, BH06.1184 and CH06.1302) on chromosomes AT06, BT06 and CH06. Red dots represent the window harboring *Hd1* genes. Red and black dashed lines represent thresholds of top 1% and 5% F_{st} values, respectively. **b** Tajima's *D* scan in 20-kb sliding windows around *Hd1*. Red dashed lines represent 95% significance thresholds. **c** Nucleotide diversity π in 20-kb sliding windows around *Hd1*.

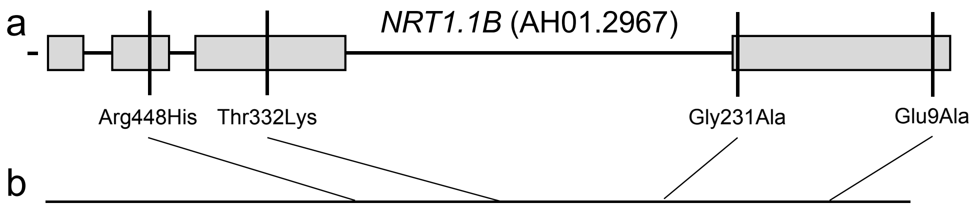

b

haplotype	C/T	G/T	C/G	T/G
Hap1	C	G	C	T
Hap2	C	G	G	G
Hap3	T	G	C	G
Hap4	T	G	G	G
Hap5	T	T	C	T
Hap6	T	T	G	G
Hap7	-	G	G	G
Hap8	T	G	-	G

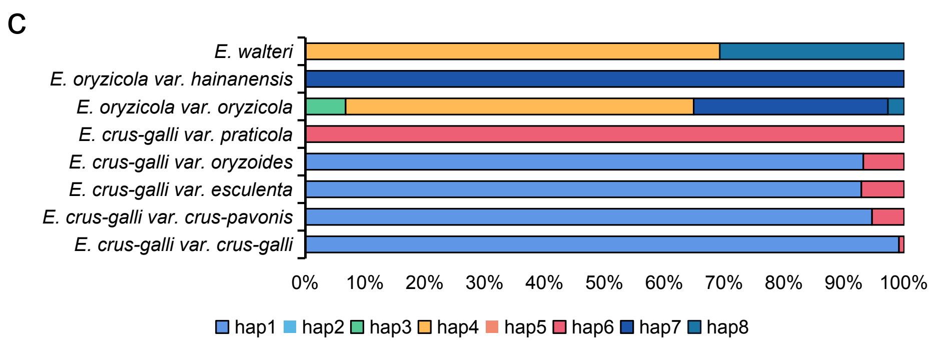

Supplementary Figure 37. Haplotype analysis of *NRT1.1B*.
a Gene structure of *NRT1.1B* (AH01.2967). Gray boxes represent exons. **b** Eight haplotypes composed by four non-synonymous SNPs. "-" represents missing. **c** Haplotype distributions in *Echinochloa* groups.

a

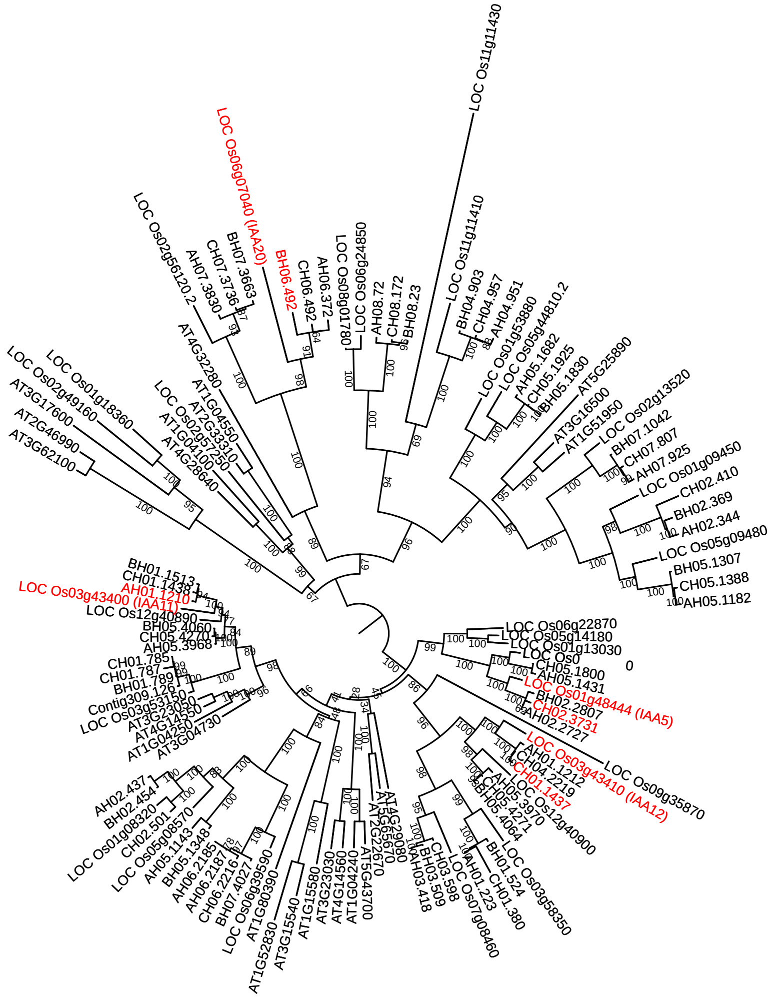

b

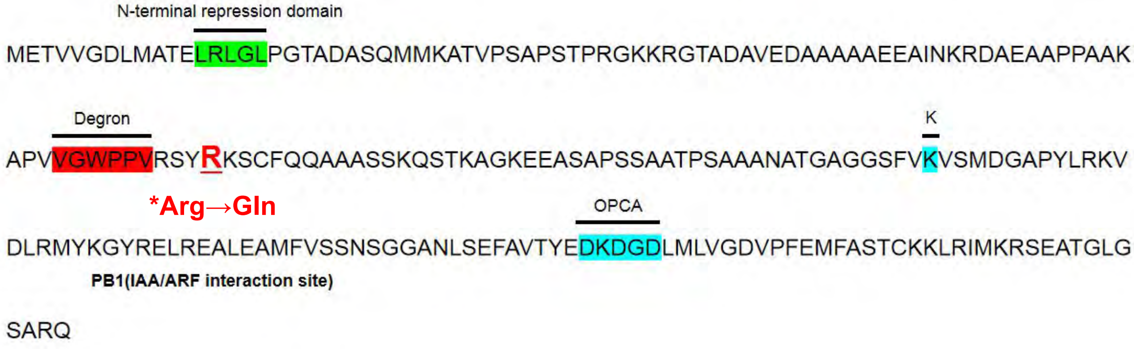

Supplementary Figure 38. Characterization of AUX/IAA genes in *E. crus-galli*.
a Phylogeny of AUX/IAA genes in *E. crus-galli* and *O. sativa* genomes. Bootstraps values are shown at the branches. **b** Structure of *EcAUX/IAA12* (CH01.1437). One mutation Arg86Gln was found in one quinclorac-resistant sample (SA88) from Brazil. Green sequence LxLxL indicates the ethylene response factor associated amphiphilic repression (EAR) motifs. The sequence in red shows the degron motif, which binds to auxin and the co-receptor TIR/AFB proteins. The K and DxD and ExD sequences (OPCA) motifs in the PB1 domain are shown in blue.

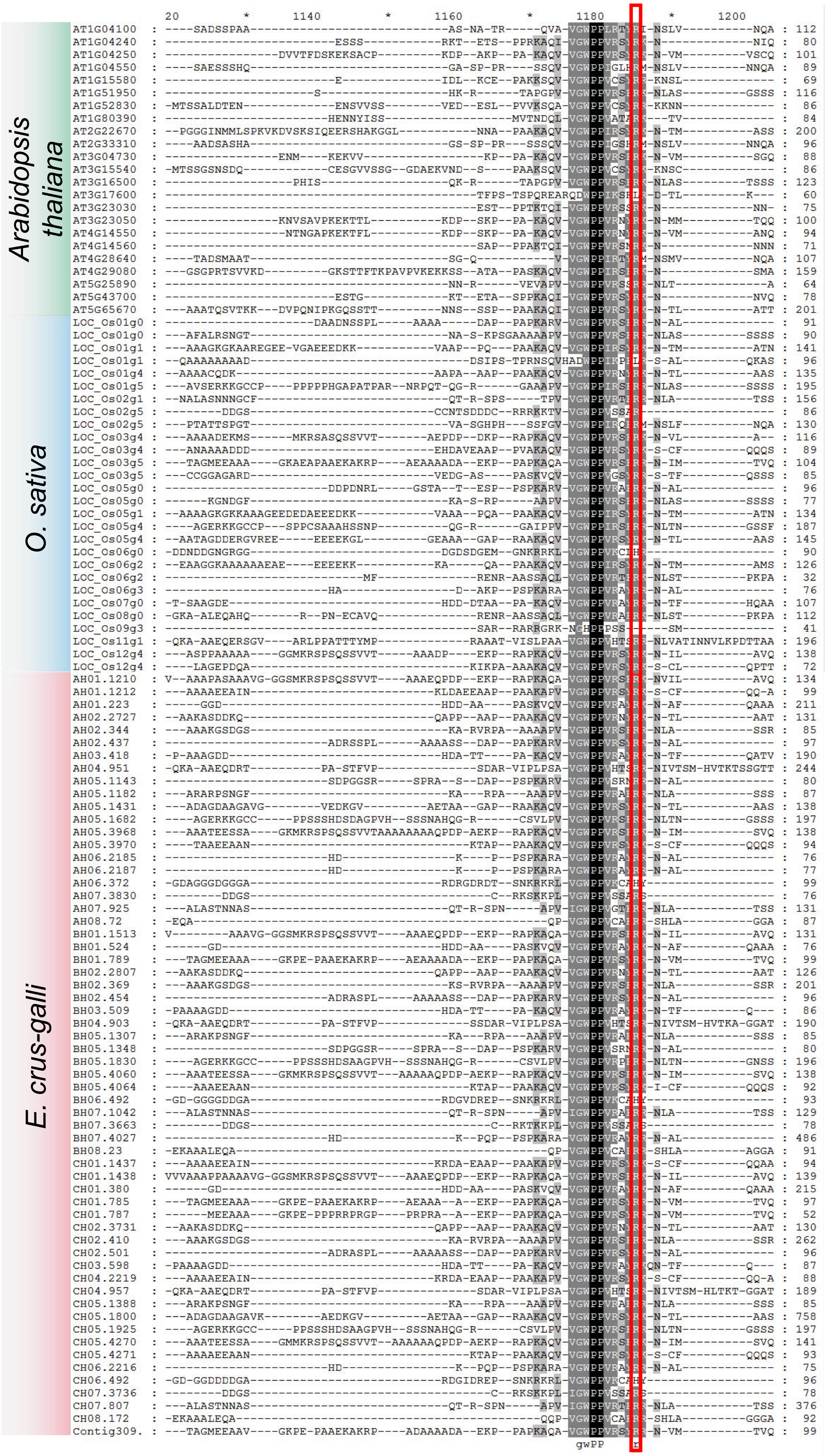

Supplementary Figure 39. Protein sequence alignment of degron regions in AUX/IAA proteins. All AUX/IAA proteins in *A. thaliana*, *O. sativa* and *E. crus-galli* are identified and aligned by MAFFT. The positions of candidate mutation (Arg86) in AUX/IAA are highlighted in red box.

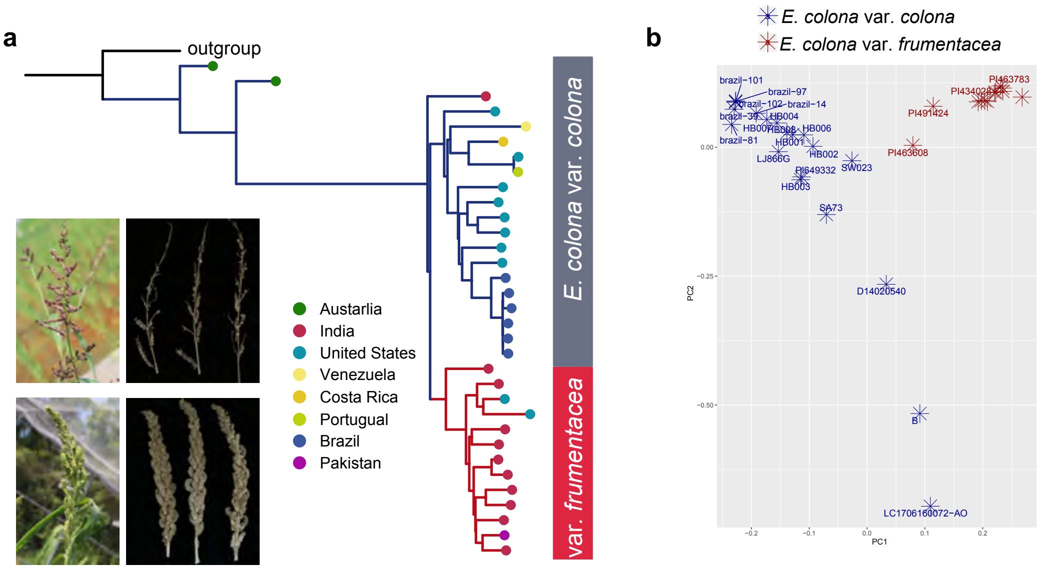

Supplementary Figure 40. Phylogeny and population structure in *E. colona*.

a A ML tree is built using 2.39 million high-quality whole-genome SNPs (MAF > 0.05 and missing rate < 0.3), with *E. crus-galli* as an outgroup. Weed (referred to as *E. colona* var. *colona*) and crop (referred to as *E. colona* var. *frumentacea*) clade are separated. Node colors represent geographical sources. Photos show the panicle differences between *E. colona* weed and crop.

b PCA plot using the first two principal components based on whole-genome SNPs.

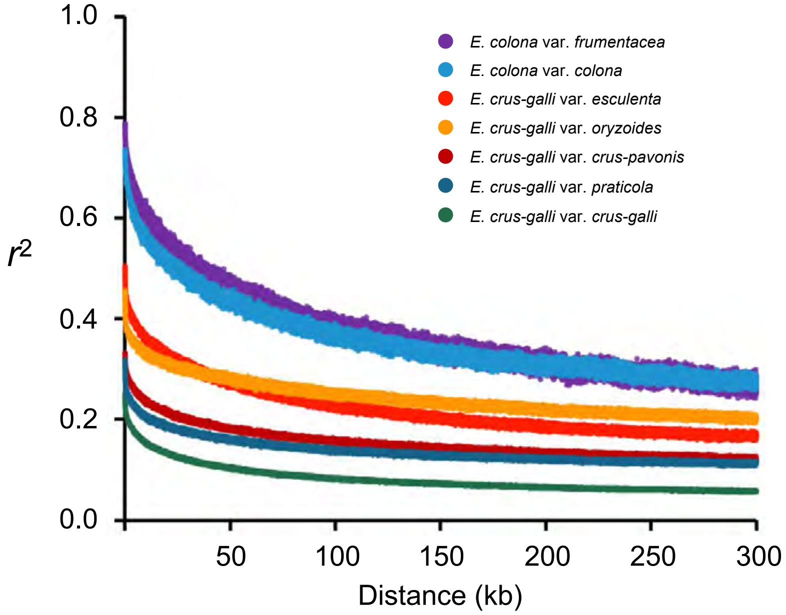

Supplementary Figure 41. Linkage disequilibrium decay in two *E. colona* and five *E. crus-galli* varieties.

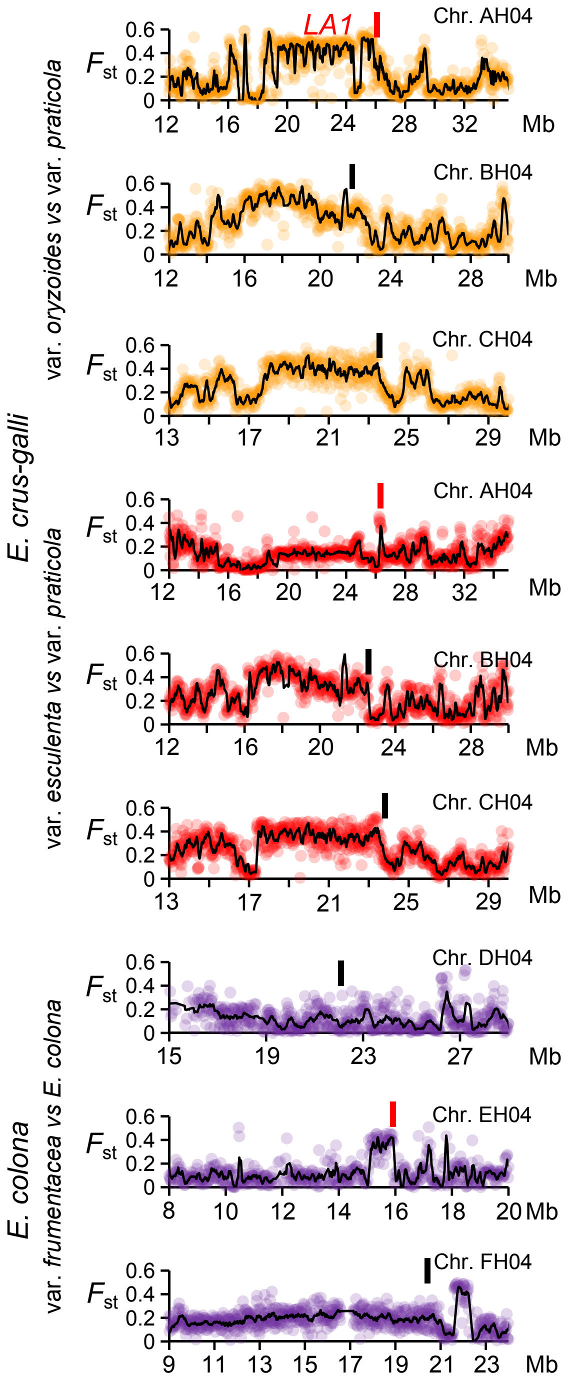

Supplementary Figure 42. Genomic differentiation (F_{st}) between barnyard grasses and millets around *LA1* on chromosomes 4.

Red blocks represent the physical positions of *LA1* genes with highly differentiated non-synonymous mutations. Black blocks represent the physical positions of *LA1* genes with no highly differentiated non-synonymous mutations.

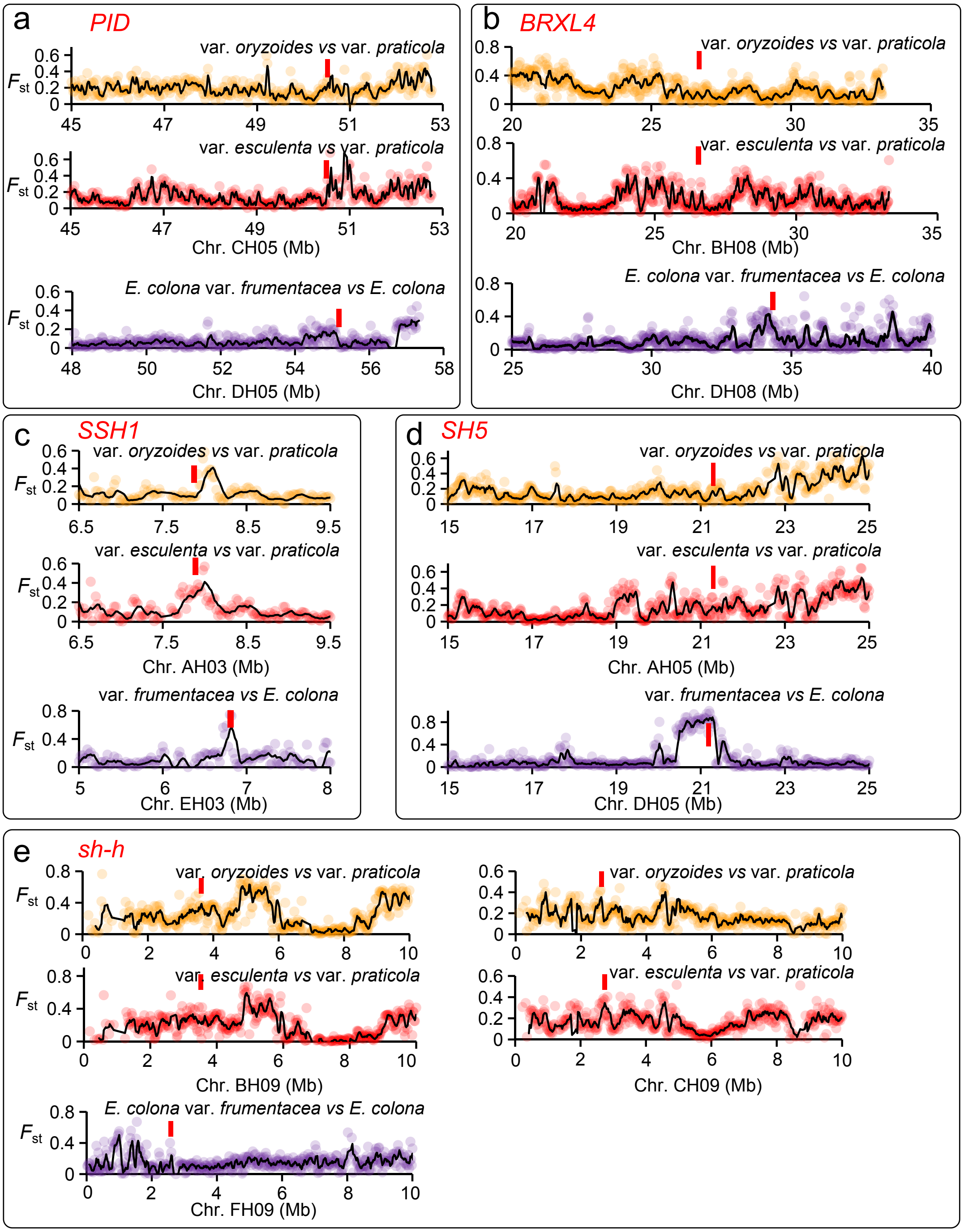

Supplementary Figure 43. Genomic differentiation (*F_{st}*) between barnyard grass and millet around genes *PID* (a), *BRXL4* (b), *SSH1* (c), *SH5* (d) and *sh-h* (e).
Red blocks represent the physical positions of genes.

Supplementary Note 1. Taxonomy and evolution of *Echinochloa*

The genus *Echinochloa*, belongs to the sub-family Panicoideae of the family Poaceae and contains approximately 50 species (Michael, 2001), which are annual or perennial grass with great morphological variations and plasticity. Some species are known as the most obnoxious weeds, mainly including *Echinochloa crus-galli*, *Echinochloa colona* and *Echinochloa oryzicola*. Additionally, at least two varieties (*E. colona* var. *frumentacea* and *E. crus-galli* var. *esculenta*) are cultivated as cereals.

Based on previous work on *Echinochloa* taxonomy from morphological, cytological and molecular marker studies (Yabuno, 1966; Yabuno, 1983; Aoki & Yamuguchi, 2009), combined with current genomic evidence, and taxonomy information from GBIF (Global Biodiversity Information Facility), iPlant.cn, Flora of China and ITIS (the Integrated Taxonomic Information System), *Echinochloa* species could be generally categorized into five groups, crus-galli group, oryzicola group, colona group, diploid group and others. Their genomic composition, living habitat, global distribution and morphology are integrated to characterize the features of each group and species.

(1) Crus-galli group

Crus-galli group includes one species *E. crus-galli*, distributed mainly in temperate regions (Yabuno, 1962). It is hexaploid with three subgenomes ($2n = 6\times = 54$). *E. crus-galli* is highly diverse in morphological and ecological features. According to phenotypes and ecological niche, this species has been classified into several botanical varieties: var. *esculenta*, var. *crus-galli*, var. *praticloa*, var. *crus-pavonis*, var. *oryzoides* and var. *formosensis* (See morphological differences in Supplementary Figs. 21 and 22).

E. crus-galli var. *esculenta* (synonymous to *Echinochloa esculenta*, *Echinochloa utilis*, *Echinochloa crus-galli* var. *utilis*) is the cultivated form that was domesticated from wild form of *E. crus-galli* about 4000 years ago and mainly cultivated in Japan, Korea, Germany, Northeast China and Yunnan Province, China (Yabuno, 1962). As a cereal, var. *esculenta* shows non-shattering grains and erect plant architecture, stouter stem and larger panicle than those of wild form. The millet was ever important in some regions of Japan as the staple food until the early Meiji Era (about 150 years ago). In addition, as a fodder this grass is considered to be superior to rice or oat regarding protein and calcium contents (Yabuno, 1971).

E. crus-galli var. *praticola*, naturally grows along roadsides or other disturbed places as a popular weed, where relative dryness prevails, and rarely in the marsh, ditch or paddy fields. The plant architecture is usually procumbent or prostrate, with slender stems and leaves, and purplish at base of culms. Panicle is narrow and the spikelets are small.

E. crus-galli var. *formosensis*, is restricted to paddy fields, abundantly occurring especially in Southeast Asia (Yabuno, 1984), is mimic to rice plant, having erect stems and leaves. Normally the spikelets are awnless.

E. crus-galli var. *crus-galli* (common name barnyardgrass), lives in paddy field, bank, ditch and marsh and mimics to rice seeding, with erect architecture, compact leaves, and

strong tillering, but variable in panicle shape, awn characteristic and spikelet size (Yabuno, 1983).

E. crus-galli var. *oryzoides*, (synonymous to *Echinochloa oryzoides*, common name early watergrass), is another rice-mimic form distributed from southern Europe to east Asia, with compact culms and stiffly erect leaves, but with long awns (~1.5cm). Particularly, spikelets are non-shattering and normally the seeds are as large as *E. oryzicola* and are larger than those of other *E. crus-galli* varieties. A race of var. *oryzoides* known as *E. macrocarpa*, has ever harvested as a crop (Rozhevits, 1937). Grains of var. *oryzoides* are used in the Caucasus for distillation of alcohol and for grinding into flour for flat cake (de Wet et al., 1982) and the market value of var. *oryzoides* almost equaled to that of barley (Rozhevits, 1934). This variety now is regarded as a paddy weed but also should be regarded as an ancient crop, based on our observation from its morphology and genomic investigation in this study.

E. crus-galli var. *crus-pavonis* (synonymous to *Echinochloa crus-pavonis*, common name Gulf cockspur), appears with robust culms, loose glabrous leaf sheaths, large, loose and nodding inflorescence, purple-tinged spikelets, and stout long awns (~1.5cm), usually decumbent at base and forming a large clump. It is usually distributed along streamsides and other damp places. Some literature considered this variety as a species or a single group compared to *E. crus-galli*, however, the genomic compositions of this variety is same as *E. crus-galli* in previous studies by molecular markers (Danquah et al., 2002) and whole-genome analysis in this study. Thus, we employed the Latin name of *E. crus-galli* var. *crus-pavonis* rather than *E. crus-pavonis* here.

(2) Oryzicola group

Oryzicola group includes several tetraploid polyploids, e.g. *E. oryzicola* and *Echinochloa wateri*. They share the same karyotype of 2n = 4× = 36, with two subgenomes, but show divergence in morphology and ecological niche (See morphological differences in Supplementary Figs. 21 and 22).

E. oryzicola (synonymous to *Echinochloa phyllopogon*, common name late watergrass) is predominantly in Northeast Asia, Europe and California in US, appearing with a high resemblance to rice in wetland rice fields with erectness of tillers and leaves. Regarding to the sterile lemma, two forms were recognized (F and C-form). F-form is short- or long-awned,but C-form are always awnless (Yabuno, 1966). In this study, we found two varieties, var. *oryzicola* and var. *hainanensis*, with different morphological features.

E. walteri (common name coast cockspur), is native in America, growing in marshes, ditches and on shorelines, including intertidal wetlands(Pennsylvania Natural Heritage Program, www.naturalheritage.state.pa.us). The awn is usually long, and the sheaths and glume surface are long-hairy.

(3) Colona group

Colona group is consisted of wild-form *E. colona* and cultivated-form *E. colona* var. *frumentacea* with a karyotype of 2n = 6× = 54. *E. colona* is widely distributed in the tropics and sub-tropics and has been domesticated into *E. colona* var. *frumentacea* as a barnyard

millet (See morphological differences in Supplementary Figs. 21 and 22).

E. colona resembles *E. crus-galli* var. *praticola* at a glance with loose architecture, slender stems and erect panicle, but usually awnless. *E. colona* var. *frumentacea* (synonymous to *Echinochloa frumentacea*) is locally known in India as Sawa or Banti, grown in the dry area as a subordinate crop to sorghum, or sometimes as a fodder or green mature crop. Non-deciduous spikelets, broad leaves, and thick stems are characteristic of cultivated var. *Frumentacea* (Yabuno, 1971).

(4) Diploid group

Diploid group contains several diploid species (2n = 2× = 18), which have been found only in Africa so far. *Echinochloa haploclada* is a perennial grass with lender long awn and shorter rachilla (than inter-node of rachis)(Supplementary Fig. 21). *Echinochloa obtusiflora* has been found only in West Africa, in dryland rice fields where African rice (*Oryza glaberrima*) was cultivated. Its distribution was connected with African rice cultivation. The plant architecture and rachis branches are erect (Yabuno, 1983).

(5) Other group

Others group contained species which are highly variable in cytotypes and genomic compositions. *Echinochloa stagnina* is distributed in Tropical Asia and Africa, and is well-known as its extreme variations in genome ploidy (Yabuno, 1970). *Echinochloa pyramidalis* is distributed in tropical African and America, and is characterized by easy budding and rooting at stem nodes. Diploid, tetraploid, hexaploid, octoploid and decaploid have been found in a collection identified as *E. pyramidalis* (Yabuno, 1983).

Generally the hybridization in *Echinochloa* is highly frequent, leading to high ploidy divergence. But the processes of polyploid formation are known a little, especially for *E. crus-galli*, *E. oryzicola* and *E. colona*, although they play significant roles in agricultural practices. Cytological relationship and molecular evidences revealed that *E. oryzicola* is a tetraploid progenitor of hexaploid *E. crus-galli* as the male donor (Yabuno, 1966; Yasuda et al., 2002; Aoki & Yamuguchi, 2008), but the third genome (female donor) is still unknown, although diploid *E. haploclada* is close genetically to the third genome with approximately divergence time of 1.6 million years (Ye et al., 2020). The hybridization time between *E. oryzicola* and the unknown diploid ancestor was estimated as 0.3 milion years ago (Ye et al., 2020). A hybrid study indicated a possibility that the genome of diploid *E. pyramidalis* may be related to one subgenome of *E. oryzicola* (Yabuno, 1976). The ancestors of *E. colona* were not known so far. The domestication of two kinds of barnyard millets were independent. *E. crus-galli* var. *esculenta* was from *E. crus-galli* wild-form in East Asia, while *E. colona* var. *frumentacea* was developed from *E. colona* in tropical Africa or India (Yabuno, 1966). Besides cultivated varieties, weeds with cultivar-like traits are found. In crus-galli group, *E. crus-galli* var. *oryzoides* shows non-shattering for spikelets, erect plant architecture and large grain size, although we consider it as weed now. In oryzicola group, *Echinochloa persistentia* shows grain non-shattering as cultivated *E. oryzicola*. It is unknown that the domesticated traits in weeds were derived from natural selection (e.g. plant architecture mimicry to rice, seed mimicry) or ancestral domesticates in ancient times which have been abandoned at present.

Supplementary Note 2. Genome scaffolding of *E. colona* assembly

E. colona is hexaploid with three subgenomes. Due to high sequence similarities among subgenomes, regular methods for Hi-C assisted scaffolding did not work well. Here we developed DipHiC to scaffold contigs to chromosome with Hi-C data by two steps (subgenome distinguishing and chromosome building). The main workflows of DipHiC applied to *E. colona*, *E. oryzicola* and *E. crus-galli* are similar, here we mainly described the species-specific analysis in *E. colona* (for detailed analysis of shared steps, please see the scaffolding of *E. oryzicola* in Supplementary Note 3). For example, the distinguishing processes of *E. colona*, *E. oryzicola* and *E. crus-galli* were different, because *E. colona* was hexaploid compared to tetraploid of *E. oryzicola* and the ancestral parental genomes could be used in distinguishing for *E. crus-galli*,while none for *E. colona*. In addition, the draft assembly of *E. crus-galli* was fragmented while the contig N50 of *E. colona* was as long as the level of sub-chromosome, which determined different strategies to distinguish subgenomes in the two hexaploid species.

(1) subgenome distinguishing

i. Mapping by a diploid genome

A diploid assembly was used to build allelic contig information and was mapped to *E. colona* contigs by splitting into 100-mers (Supplementary Fig. 2a). Mapping depth was calculated in windows of 1 Mb and 100 kb. If more than one peak could be clearly observed, this diploid assembly has the potential to distinguish subgenomes of the hexaploid genome, because it indicates different genetic distances from diploid to each subgenome. In this case, when 100-mer split from diploid *E. haploclada* were mapped to hexaploid *E. colona* contigs, three peaks were observed in mapping depth distribution, which meant *E. haploclada* could provide useful information to subgenome distinguishing (Supplementary Fig. 2b).

ii. Allelic contig (Actg) information construction

Subgenome synteny is informative in distinguishing subgenomes, in spite of possible homeologous exchanges. The second step is to obtain allelic contig information. Contigs were annotated by gmap with *E. haploclada* CDS sequences. Syntenic relationships among contigs were determined by counting shared gene number (e.g. greater than 50). Firstly we got pairwise relationship of contigs (Supplementary Fig. 2c). Syntenic contigs are mutually exclusive as illustrated in (i) (black lines with double arrows. e.g. contig1 to contig 2, contig6 to contig7) because they are not likely from the same subgenome. Based on direct mutually exclusive relationships, indirect networks could be further expanded (Supplementary Fig. 2c). For three subgenomes in the assembly, if contig1, contig2 and contig3 were mutually exclusive, we could infer that they belonged to three subgenomes, respectively. Here contig5 was exclusive with contig1 and contig2, it was reasonable to determine that contig3 and contig5 were from a shared subgenome (illustrated as red lines in Supplementary Fig. 2c ii). Finally, contig4 and contig5 were mutually exclusive, because contig3 and contig4 were inter-subgenome and conig3 and contig5 were within one subgenome (illustrated as blue lines with double arrows in Supplementary Fig. 2c iii).

iii. Three rounds of clustering

According to the depth when 100-mers were mapped to the draft contigs, contigs (first column in Supplementary Fig. 2d) were assigned into *cluster1*, *cluster2*, *cluster3* and unknown cluster (*uncluster*; second column in Supplementary Fig. 2d). Based on the allelic contig information, the clustering was corrected and reassigned (third column in Supplementary Fig. 2d). For example, for contig ptg000001l, in the 1st-round it was categorized as *uncluster*, while its allelic contig information supported that it belongs to *cluster3*, thus this contig was assigned in *cluster3*. For contig ptg000002l, the first clustering it was in *cluster1* and the allelism also supports the assignment, thus this contig was assigned in *cluster1* after two rounds clustering. For contig ptg00021l, although the first clustering it was in *cluster1*, no allelic information further supported this clustering, and thus this contig was re-assigned in *uncluster*. The 3rd-round clustering was based on inter-contig interactions or linkages by Hi-C data or long-insert mate-paired reads. We counted the interactions among contigs, and then calculated the interactions to three clusters or subgenomes (according to the clustering in 2nd-round) for each contig and corrected the clustering based on interaction bias (columns 6-8 in Supplementary Fig. 2d). For example, for contig ptg000001l, this contig showed significantly more interactions to *cluster3* and its clustering in 2nd-round was *cluster3*, thus we finally confirmed the assignment of this contig to *cluster3*. For contig ptg000013l, no interaction bias was found to three clusters, thus finally clustering of this contig was not resolved. Finally, contigs were distinguished into three clusters or subgenomes. See more details about three rounds of clustering at Supplementary Note 3.

(2) Chromosome building

The same pipeline as *E. oryzicola* chromosome building. See details in Supplementary Note 3.

Supplementary Note 3. Genome scaffolding of *E. oryzicola* assembly

Previous assembling of *E. oryzicola* genome with Pacbio long reads yielded a draft assembly of ~954 Mb, with contig N50 size of 1.9 Mb (Ye et al., 2020). Analysis based on collinear homologous genes between two subgenomes revealed the divergence event between subgenomes at 4.6 mya and merge at 1.9 mya. We first used popular methods to scaffold contigs into chromosomes, however, the performances were not good as expected. Here DipHiC was used in HiC-assisted genome scaffolding of *E. oryzicola*, by subgenome distinguishing and chromosome building, which could be complementary to other methods or software in some complex polyploid genome assembly.

(1) Subgenome distinguishing

To assign contigs into two subgenomes, a high-quality diploid relative genome and CDS annotation files were used. For *E. oryzicola*, *E. haploclada* was selected due to its close phylogenic relationship and high-quality genome assembly.

i. Clustering based on depth

Firstly, whole genome sequences of *E. haploclada* were split into 100-mer short reads, and these reads were mapped to the *E. oryzicola* contigs using BWA. The mapping depth was counted using samtools, and average depth for each contig was calculated. To determine the thresholds to split, a sliding-window average depth file should be calculated with proper window size (e.g.1 Mb, 500 kb or 200 kb). Two peaks would be observed representing two subgenomes (Supplementary Fig. 5a i). Here, we calculated depth distribution in contig with different window sizes of 1 Mb, 500 kb, 200 kb, 100 kb and 50 kb. In most plots, two peaks were observed, except window sizes less than 100 kb, which implied that this strategy would be not efficient for draft genomes with low continuity. Finally, two peaks when depth equaled 13.35 and 36.26 were chosen as average mapping depth for two subgenomes.

Here R1 and R2 were used to limit the depth distribution boundaries of two subgenomes (Supplementary Fig. 5a i). Contigs with average depth less than (P1+R1*ΔP) were assigned into *cluster1* and contigs with depth greater than (P2-R2*ΔP) were *cluster2*, where ΔP=P2-P1. To determine the optimal R1 and R2 values (higher clustering coverage and low error rate), *Brassica* genomes were used as test dataset. *Brassica juncea* genome contains two subgenomes (BjuA and BjuB), and BjuA shows closer relationship to diploid C (*Brassica oleracea*), which formed a similar topology as *E. oryzicola* and *E. haploclada* (Yang et al., 2016).

100-mers from *B. oleracea* genome were mapped against *B. juncea* genome and depth were calculated in 5Mb, 2Mb, 1Mb, 500kb and 200kb-window segments, respectively. Considering the gaps region would influence mapping and bring noises of low depth, segments with more than 30% Ns (gaps) were removed. Two peaks were observed, in line with the real distribution of subgenome segments average depth, and the subgenome with higher depth usually showed broader ranges, which may caused the clustering errors if just based on depth information. Here, peak depths were 5.5 and 41.6 for subgenome B and A . R1 and R2 values from 0 to 0.9 with a step of 0.05, the clustering error rates were calculated. With the increasing of the R value, accuracy decreased obviously for *cluster1* whose average depth was lower, while accuracy was more robust for *cluster2*. To ensure the accuracy of clustering and also to improve the coverage as high as possible, here R1 was assigned as 0.10 and R2 was 0.55 for further test, and accuracy of first round

clustering was 99.64% for cluster1 and 98.62% for cluster2, and 92.2% of genome was covered.

ii. Clustering based on contig allelism

To construct allelic contig information (allelism), contigs were annotated by diploid relative CDS annotation using gmap. Counting the shared loci annotated, allelic contigs table was built. Five columns in this allelic contig table represented contig A, loci annotated in contig A, contig B, loci annotated in contig B and shared loci annotated in both contig A and B. Shared loci number and its proportion in annotated loci within each contig were used to filter the allelic information.

In first-round clustering, contigs were clustered into three clusters (*cluster1*, *cluster2* and *uncluster*)(Supplementary Fig. 5a ii). Allelic contigs normally would not belong to the same cluster. As Supplementary Fig. 5a ii showed, (a), contig1 was assigned into *cluster1*, and its allelic contig was contig10, a member in *cluster2*, thus the clustering of contig1 was supported by allelism (pass). (b), contig2 was in *cluster2*, its allelic contigs were contig9 and contig67, belonging to *cluster2* and *cluster1*. Considering the shared loci number, contig9 was more likely to be allelic. While both contig2 and contig9 were in *cluster2*, the clustering was not supported (doubt). c, contig3 was in *uncluster*, and its allelic contig contig8 was in *cluster1*, thus contig3 would be clustered into *cluster2* (pass). d, contig4 was in *cluster1*, while no allelic contigs were identified, thus it would be moved to *uncluster* (noActg). In this way, clustering of allelic contigs would be more reliable than first-round results, in spite of the loss of genome coverage.

In *Brassica* test, contig allelism was created by filtering with shared loci number ≥5 and proportion to the smaller annotated loci number ≥20%. After second-round clustering, 86.4% of contigs or segments were clustered into cluster 1 and 2, with 98.9% and 99.2% accuracy for two clusters.

iii. Clustering based on interaction

After two rounds clustering, still 13.6% segments remained unclustered in *B. juncea*. Third-round clustering was to rescue these segments or contigs, based on linkage information from mate-pair reads, Hi-C data or other long-distance interaction signals. A 10-kb mate-paired library from previous study (Yang et al., 2016) was used to investigate linkages. Via Bowtie2, clean reads were mapped to the segments, and after removing adapters and duplication, the links information was obtained and linkages among segments were counted. For each segment, links were categorized as links to *cluster1* (L1), *cluster2* (L2) and *uncluster* and then bias index α was calculated as L2/L1 for each segment. Also average α for *cluster1* and *cluster2* were calculated ($\alpha 1$ and $\alpha 2$). Similar to first-round clustering, unclustered segments would be clustered by their bias indexes. If the index was less than ($\alpha 1 + R^* \Delta\alpha$) (where $\Delta\alpha = \alpha 2 - \alpha 1$), the category was *cluster1*; if greater than ($\alpha 2 - R^* \Delta\alpha$), the category was *cluster2*; if either not, this segment still remained unclustered (Supplementary Fig. 5a iii). After third-round clustering, most unclustered segments would be categorized. To further rescue these unclustered segments, more iterations of third-round clustering could be carried by re-calculating linkages and re-clustering.

In *Brassica* test, after 3rd-round clustering, the clustered coverage reached 97.4% (only 86.4% in 2nd-round). One iteration of 3-round was run and finally 98.2% of segments were clustered into cluster1(315 segments, accuracy 99.0%) and cluster2 (290 segments,

99.3%). For *E. oryzicola*, a similar pipeline was used: 1st depth clustering, 2nd allelism clustering, 3rd linkage clustering, 4th linkage iteration, 5th allelism clustering, 6th linkage clustering, 7th linkage iteration, 8th linkage iteration (in 1st-round, P1=13.35, P2=36.26, R1=0.10, R2=0.50; in 2nd-round, *E. haploclada* cds annotation was used to build allelic contig table with shared loci number > 5 and shared proportion > 20%; in 3rd-round, R was set as 0.10). Finally 97.9% size of *E. oryzicola* contigs (945.5 Mb) were categorized as *cluster1* (505.8 Mb) and *cluster2* (419.8 Mb), and 19.8 Mb contigs were not distinguished.

(2) Chromosome building

Most contigs have been assigned into two subgenomes. Draft genome and Hi-C mapping data then were split to two sets of subgenomes. For each subgenome, contigs could be further grouped, ordered and orientated using regular pipelines for diploids, e.g. LACHESIS, juicer+3d-dna, SALSA and AllHiC. AllHiC and Lachesis were used to anchoring contigs with each subgenome. The results were not good as expected (Supplementary Fig. 5c), may be due to too much signal loss in subgenome distinguishing. To solve this, DipHiC chromosome building pipeline was built (Supplementary Fig. 5b).

Firstly, directional interaction intensity was calculated for each contig (Supplementary Fig. 5b i). Links between source contig and other target contigs were counted, then interaction intensity equaled link number divided by length of the target contig. Secondly, low-intensity and discordant inter-contig linkages were removed (Supplementary Fig. 5b ii). Linkages with links less than 10 and intensity less than 10 (10 links per Mb) between contigs were filtered out. Based on previous annotation by *E. haploclada* CDS, long contigs (at least 10 loci could be annotated and 40% of loci belong to one chromosome in *E. haploclada*) were pre-assigned into groups. For each source contig, linkages were ranked by interaction intensity, considering chromosome rearrangements, two pre-assigned groups were allowed to be linked by the source contig.

Thirdly, a directional interaction network was constructed among contigs. In the diagram of Supplementary Fig. 5b iii, when contig1 was regarded as a source, most of its target contigs were pre-assigned in group “red”, here contig1 was categorized as “red” . Meanwhile, when contig1 was a target, most of its source contigs were “red”, then contig1 was “red”. Only the categories when a contig was a souce and a target, were consistent, the category was reliable. Contig4 was pre-assigned as “green”, while the category when contig4 was either a source or target, was “red”, finally contig4 was grouped in “red”, which did not determined by its initial pre-assignment. In this way, contigs were assigned to different groups. It was worth noting that *E. haploclada* showed same chromosome number with subgenome in *E. oryzicola*. The diploid relative should be close genetically to subgenomes. If a diploid with discordant chromosome number was used, how to group has not been explored.

Contigs within each chromosome were ordered and orientated by module optimize of AllHiC (Supplementary Fig. 5b iv). Finally, 497.7 Mb of *cluster1* (AT, total size 505.8 Mb) and 407.0 Mb of *cluster2* (BT, total size 419.8 Mb) were anchored to 9 and 9 chromosomes in subgenome A and B, respectively, totally representing 95.7% of whole genome sequences (Supplementary Figs. 7 and 8).

Supplementary Note 4. Genome scaffolding of hexaploid *E. crus-galli* assembly

(1) Contig correction

Due to the nature of hexaploidy and low continuity of *E. crus-galli* draft assembly, before HiC scaffolding, extra contig correction was performed to ensure the assembly accuracy (Supplementary Fig. 6a). Diploid genome *E. haploclada* and two subgenomes from tetraploid *E. oryzicola* were used to distinguish subgenomes of hexaploid *E. crus-galli*, by mapping 100-mers to *E. crus-galli* contigs. For each contig, average depths were calculated for three ancestral subgenomes as d(CH), d(AH), d(BH) and summed as SUM(d). When max[d(CH), d(AH), d(BH)]/SUM(d) less than 0.7, this contig was regarded as discordant, which was putatively caused by misjoin of two or more genomic segments from different subgenomes. Putative break-points were scanned by 5-kb sliding window depth. Considering the presence of homeologous exchanges (HEs) in hexaploid, Hi-C and 20-kb mate-paired reads were mapped against contigs and links were counted within flanking regions (here 40 kb leftward to 25 kb rightward) of putative break-points. Break-points with at least 50 links for 20 kb mate-paired library or 5 links for Hi-C library were considered as a candidate HE. Otherwise, the break-points were junctions of misjoins and contigs were split there (Supplementary Fig. 6a).

(2) Subgenome distinguishing

After contig correction, 100-mers from three ancestral subgenomes were mapped to modified contigs. Categories of contigs were determined by the average depths of three ancestral subgenome only when the maximum depth proportion was greater than 0.7. After this, 760, 522, 716 and 2025 contigs were assigned as AH, BH, CH and UC(unclustered), representing 449, 320, 401 and 170 Mb in size. To cluster UC, Hi-C links between contigs were calculated. Due to high similarities among three subgenomes, many noisy links was observed. Although it was hard to distinguish subgenomes just based on single contigs, subgenomes could be divided overall from target subgenome proportions. For one contig, links to other contigs could be counted and we would know subgenome proportion of links to this contig (Supplementary Fig. 6b). Average linkage proportions for three target subgenomes were featured as center points (P1, P2, P3) by calculating the median proportions of contigs (length > 0.1 Mb and links number to three subgenomes > 10) labeled as each subgenome, respectively. Euclidean distance was calculated between proportion triplet for each contig to three center points, respectively (Supplementary Fig. 6c). Only when the minimum distance shorter than 2/3 of the median of the three distances, this contig was assigned as the cluster of the closest center point. Finally, in AH, BH and CH, 99.2%, 95.4% and 98.0% of contigs in size were consistently classified in two rounds. For UC, 89.1% of contigs in size were categorized. Overall after two rounds clustering, 2487 contigs were clustered into three subgenomes with 496.6 Mb in AH, 410.7 Mb in BH and 432.4 Mb in CH.

(3) Chromosome building

Following the pipeline in *E. oryzicola* subgenome contig ordering and orientation (See Supplementary Note 2), long contigs were firstly pre-assigned and linkage intensities among contigs were calculated. AllHiC was used to order and orientate contigs within each subgenome. Finally, 95.3% (473.2/496.6 Mb), 97.2% (399.0/410.7 Mb) and 96.4% (416.8/432.4 Mb) contigs were anchored to 9, 9 and 9 pseudo-chromosomes, respectively. In total, 1289.0 Mb (96.2%) of whole genome sequences (size of 1339.6 Mb) were assigned into 27 pseudo-chromosomes (Supplementary Figs. 7b and 8a).

Supplementary Note 5. Herbicide sensitivity bioassays in Brazilian and Italian barnyard grass populations

(1) Brazilian population

Plants were grown in 200 mL pots in greenhouse at 25°C±5°C and were sprayed in three- to four-leaf stage. Pots were maintained flooded. The herbicides were sprayed in an automated spray chamber (Generation III Research Sprayer, De Vries Manufacturing Inc), using a TJ8002E spray nozzle, with a constant pressure of 42 lb pol^{-2} and speed of 1.16 m s^{-1}, resulting in a volume of 200 L ha^{-1}. The herbicides treatments were an untreated check and the Acetolactate Synthase (ALS) inhibitor herbicide Imazethapyr (Pivot 100 SL, 100 g L^{-1}, BASF S.A.) at 100 g ha^{-1} with the adjuvant Dash HC (5% of oleic acid, 22.5% of polyoxyalkylene fatty alcohol phosphate esters, and 37.5% of fatty acid methyl esters, BASF) at 0.5% v/v, the Acetyl Coa Carboxylase (ACCase) inhibitors herbicides fenoxaprop-p-ethyl (Starice®, 69 g L^{-1}, Bayer) at 69 g ha^{-1} with adjuvant Aureo® (soybean oil methyl ester, 720 69 g L^{-1}, Bayer) at 0.5% v/v and cyhalofop-butyl (Clincher, 180 g L^{-1}, Dow Agrosciences) at 360 g ha^{-1} with Veget'Oil (fatty acids vegetable oil, Oxiquímca) at 2.0 L ha^{-1}, the auxin-type herbicide quinclorac (Facet, 500 g L^{-1}, BASF) at 375 g ha^{-1} plus the adjuvant Assist (mineral oil) at 0,5% v/v, and the Enol Pyruvyl Shikimate Phosphate Synthase (EPSP) inhibitor glyphosate (Roundup Original, 356 g L^{-1}, Monsanto) at 1080 g ha^{-1}. The experiment was performed at completely randomized design with four repetitions. The evaluation occurred at 7, 14 and 21 days after treatment (DAT). Resistance was assigned to plants with less than 20 % of herbicide symptom in comparison with untreated check at 7 and 14 DAT and absence of leaf necrosis at 21 DAT.

(2) Italian population

Whole-plant sensitivity assays with ALS and ACCase inhibitors were performed in the greenhouse at Legnaro (PD), Italy. A reference population, sensitive to all the herbicides assayed was included in the experiment (07-16L). To break dormancy, seeds were chemically scarified in concentrated sulfuric acid (96%) for twenty minutes, carefully rinsed with water and sown in plastic boxes containing agar medium 0.6% and KNO_3 0.2%. Boxes were then placed in a germination cabinet at 26°C/16°C (day/night) under neon tubes providing a Photosynthetic Photon Flux Density (PFFD) of 15-30 μmol m^{-2} s^{-1} with a 12h photoperiod. After germination, *Echinochloa* seedlings were transplanted into plastic trays (32.5 × 26.5 × 9.5 cm) containing a standard potting mix (60% silty loam soil, 15% sand, 15% perlite, 10% peat). For each population and herbicide rate, two trays (replicates) with 15-20 plants were done and they were placed in the greenhouse where the temperature ranged from 16 to 19°C and from 26 to 33 °C night/day, respectively. When the seedlings reached the 3-4 true leaf stage, herbicides were applied using a bench sprayer equipped with three flat fan (extended range) hydraulic nozzles (Teejet, 11002) delivering 300 L ha^{-1} at a pressure of 215 kPa. The following herbicides were used at the recommended field rate: penoxsulam (ViperTM®, 20 g a.i. L^{-1}, Corteva Agriscience, Cremona, Italy), applied at 40 g.a.i. ha^{-1}; profoxydim (Aura®, 200 g a.i. L^{-1}, BASF, Cesano Maderno, Italy) applied at 100 g a.i. ha^{-1} with the surfactant Dash HC at 0.8 % v/v. Four weeks after treatment, the number of surviving plants was assessed.

Supplementary Note 6. NB-ARC gene family copy number estimation

Comparison of gene family size was usually based on functional domain annotations of predicted protein sequences in *de novo* assemblies. However, a single reference genome was not sufficient to investigate the copy number diversity of a species or a population, due to incompleteness of assembly, different approaches to gene annotation and others. Pan-genome analyses by *de novo* assembling multiple genomes in a species or population have provided good solutions to analyze gene family copy number variations, but the cost of pan-genomes is still high so far. Here we built a simple but effective pipeline called CNVings (Gene family Copy Number Variation inferring from next-generation sequencing) to compare gene family copy number difference in population level using NGS re-sequencing data. CNVings estimated the copy number of a specific gene family by measuring the relative reads abundance compared to reference genome that the NGS short reads were mapped to. Taken NB-ARC gene family as an example, the positions of all NB-ARC genes in the reference genome were obtained by whole-genome gene function annotations inferred by InterproScan (*e* value < 1e-10). The abundance of NB-ARC gene family across the whole genome was measured by the sum of average depthes of all loci using Mosdepth. The copy number was then standardized by the average mapping depth across the whole genome.

Obviously, sequencing depth would influence the estimations. Here taking rice as an example, when Illumina short paired-end reads of Nipponbare with different sequencing depth (NCBI run: SRR1178954; sequencing platform: Illumina HiSeq 2000) were mapped to the reference genome (IRGSP v1.0), the copy number gradually converged since 10× depth. Base on this, accessions with less than 10× sequencing depth were removed in following analysis.

To confirm the effectiveness of this pipeline, we compared the relative copy number to reference genome (IRGSP v1.0) inferred from CNVings and *de novo* assemblies of five platinum standard genomes of rice (variety CHAO MEO::IRGC80273, NANGKA::IRGC19961, ARC 10497::IRGC12485, PR 106::IRGC53418 and LIMA::IRGC81487) (Zhou *et al.*, 2020). To rule out the influences of gene annotation approaches, we firstly re-annotated the five and IRGSP genomes by RepeatMasker and Fgenesh. The predicted genes were further annotated by InterproScan (*e* value < 1e-10), and the fold changes of NB-ARC gene copy numbers in five genomes compared to IRGSP were calculated. The NGS paired-end reads with average depth of 10× of five rice varieties (Illumina Hiseq X10) were mapped to IRGSP, and CNVings estimated the fold change of NB-ARC copy number relative to IRGSP in each variety. The results based on NGS and *de novo* assemblies showed highly consistent (R^2=0.89), although the fold change in individual was not exactly the same. Briefly, this method could be used to measure the relative copy number size among populations.

Supplementary Reference

1. Michael, P. The taxonomy and distribution of *Echinochloa* species (barnyard grasses) in the Asian-Pacific region, with a review of pertinent biological studies. In: Proceedings of the 18th APWSS Conference (Beijing, China, 28 May-2 June 2001). Standard Press of China, Beijing, 57-66 (2001).
2. Yabuno, T. Biosystematic study of the genus *Echinochloa*. *Jpn J. Bot*. **19**, 277–323 (1966).
3. Yabuno, T. Biology of *Echinochloa* species. In taxonomy and distribution of *Echinochloa* species with special reference to their occurrence as weeds of rice, pp. 307-318. Los Banos, Philippines: International Rice Research Institute. (1983).
4. Aoki, D. & Yamaguchi, H. *Oryza sh4* gene homologue represents homoeologous genomic copies in polyploid *Echinochloa*. *Weed Biol. Manag.* **9**, 225–233 (2009).
5. Yabuno, T. Cytotaxonomic studies on the two cultivated species and the wild relatives in the genus *Echinochloa*. *Cytologia*. **27**, 296-305 (1962).
6. Yabuno, T. A note on barnyard millet. *SABRAO Newsletter* **3**, 43-45 (1971).
7. Yabuno, T. A biosystematic study on *Echinochloa oryzoides* (Ard.) Fritsch. *Cytologia* **49**, 673-678 (1984).
8. Rozhevits, R. Y. Grasses: an introduction to the study of fodder and cereal grasses. Translated from Russian (1980). Indian National Scientific Documentation Centre, New Delhi (1937).
9. de Wet, J.M.J. Domestication of Sawa millet (*Echinochloa colona*). *Econ. Bot.* **37**, 283-191 (1982).
10. Rozhevits, R. Y. Grasses-Gramineae Juss. In Flora of the U.S.S.R. Vol. 2, V. L. Komorov, ed, p. 1-622. Translated from Russian (1963). Israel Program for Scientific Translations, Jerusalem (1934).
11. Danquah, E. Y., Johnson, D. E., Riches, C., Arnold, G. M. & Karp, A. Genetic diversity in *Echinochloa* spp. collected from different geographic origins and within rice fields in Cote d'Ivoire. *Weed Res.* **42**, 394–405 (2002).
12. Yabuno, T. Biosystematics of *Echinochloa* stagnina (Retz.) P. Beauv., cytological relationship between the 12- and 14-ploid strains. *Genetica* **41**, 311-315 (1970).
13. Yasuda, K., Yano, A., Nakayama, Y. & Yamaguchi, H. Molecular identification of *Echinochloa oryzicola* Vasing. and *E. crus-galli* (L.) Beauv. using a polymerase chain reaction-restriction fragment length polymorphism technique. *Weed Biol. Manag.* **2**, 11–17 (2002).
14. Aoki, D. & Yamaguchi, H. Genetic relationship between *Echinochloa crus-galli* and *Echinochloa oryzicola* accessions inferred from internal transcribed spacer and chloroplast DNA sequences. *Weed Biol. Manag* **8**, 233–242 (2008).
15. Ye, C. Y. et al. The genomes of the allohexaploid *Echinochloa crus-galli* and its progenitors provide insights into polyploidization-driven adaptation. *Mol. Plant* **13**, 1298–1310 (2020).
16. Yabuno, T. Cytological relationship between the Kentan diploid strain of *Echinochloa pyramidalis* and *E. oryzicola*. *Cytologia* **41**, 679-684 (1976).
17. Yang, J. et al. The genome sequence of allopolyploid *Brassica juncea* and analysis of differential homoeolog gene expression influencing selection. *Nat. Genet*. **48**, 1225–1232 (2016).
18. Zhou, Y. et al. A platinum standard pan-genome resource that represents the population structure of Asian rice. *Sci. Data* **7**, 1–11 (2020).
