# Supplementary material for: Genomic insights into the evolution of Echinochloa species as weed and orphan crop
Source: Nat Commun. 2022 Feb 3;13:689. doi: 10.1038/s41467-022-28359-9 (PMC8814039; doi:10.1038/s41467-022-28359-9)
Supplement: Supplementary file 3 — Description of Additional Supplementary Files [file 41467_2022_28359_MOESM3_ESM.pdf]

Description of Additional Supplementary Files

File Name: Supplementary Data 1
Description: Metadata of 40 plant genomes used in phylogeny and gene family analyses in this study.

File Name: Supplementary Data 2
Description: QuIBL Results in various triplets.

File Name: Supplementary Data 3
Description: Metadata of 737 re-sequenced Echinochloa accessions in this study.

File Name: Supplementary Data 4
Description: Inferred parameters under the final joint model for all six populations. Only migrations whose estimations are greater than 1e-5 are shown in Fig. 2e.

File Name: Supplementary Data 5
Description: Genes within highly differentiated genomic regions (top 5% of F_{st} values, 0.41) between *E. crus-galli* var. *crus-galli* and var. *praticola*.

File Name: Supplementary Data 6
Description: Genes within highly differentiated genomic regions (top 5% of F_{st} values, 0.27) between high-latitude and low-latitude groups in *E. oryzicola*.

File Name: Supplementary Data 7
Description: Genes around GWAS outlier loci associated with flowering time in 461 individuals of *E. crus-galli*.

File Name: Supplementary Data 8
Description: ALS mutation spectrum in the global *Echinochloa* plants collection

File Name: Supplementary Data 9
Description: Herbicide resistance analysis of Brazilian and Italian barnyard grass populations.

File Name: Supplementary Data 10
Description: Resequencing data of Coix, sorghum and rice accessions used in NB-ARC gene copy number inference.

File Name: Supplementary Data 11
Description: Allele frequency and effects of non-synonymous variations in *Echinochloa* cultivated and weed/wild populations.

File Name: Supplementary Data 12
Description: Genes within highly differentiated genomic regions (top 5% of F_{st} values, 0.43) between barnyard millet *E. crus-galli* var. *esculenta* and var. *praticola*.

File Name: Supplementary Data 13
Description: Genes within highly differentiated genomic regions (top 5% of F_{st} values, 0.46) between *E. crus-galli* var. *oryzoides* and var. *praticola*.

File Name: Supplementary Data 14
Description: Genes within highly differentiated genomic regions (top 5% of F_{st} values, 0.43) between wild and cultivated *E. colona*.
