# Supplementary material for: Genomic insights into the evolution of Echinochloa species as weed and orphan crop
Source: Nat Commun. 2022 Feb 3;13:689. doi: 10.1038/s41467-022-28359-9 (PMC8814039; doi:10.1038/s41467-022-28359-9)
Supplement: Supplementary file 18 — Reporting Summary [file 41467_2022_28359_MOESM18_ESM.pdf]

nature portfolio

Corresponding author(s): Chu-Yu Ye
Last updated by author(s): Jan 10, 2022

Reporting Summary

Nature Portfolio wishes to improve the reproducibility of the work that we publish. This form provides structure for consistency and transparency in reporting. For further information on Nature Portfolio policies, see our Editorial Policies and the Editorial Policy Checklist.

Statistics

For all statistical analyses, confirm that the following items are present in the figure legend, table legend, main text, or Methods section.

n/a	Confirmed	
☐	☒	The exact sample size (*n*) for each experimental group/condition, given as a discrete number and unit of measurement
☐	☒	A statement on whether measurements were taken from distinct samples or whether the same sample was measured repeatedly
☐	☒	The statistical test(s) used AND whether they are one- or two-sided<br>*Only common tests should be described solely by name; describe more complex techniques in the Methods section.*
☒	☐	A description of all covariates tested
☐	☒	A description of any assumptions or corrections, such as tests of normality and adjustment for multiple comparisons
☐	☒	A full description of the statistical parameters including central tendency (e.g. means) or other basic estimates (e.g. regression coefficient) AND variation (e.g. standard deviation) or associated estimates of uncertainty (e.g. confidence intervals)
☐	☒	For null hypothesis testing, the test statistic (e.g. *F*, *t*, *r*) with confidence intervals, effect sizes, degrees of freedom and *P* value noted<br>*Give P values as exact values whenever suitable.*
☒	☐	For Bayesian analysis, information on the choice of priors and Markov chain Monte Carlo settings
☒	☐	For hierarchical and complex designs, identification of the appropriate level for tests and full reporting of outcomes
☐	☒	Estimates of effect sizes (e.g. Cohen's *d*, Pearson's *r*), indicating how they were calculated

Our web collection on statistics for biologists contains articles on many of the points above.

Software and code

Policy information about availability of computer code

Data collection	DNA sequencing of E. colona was performed using Pacbio HiFi platform (CCS mode) and corresponding softwares from the manufacturers. HiC sequencing of E. colona, E. crus-galli and E. oryzicola was performed using Illumina HiSeq 2500 platform (150bp paired-end reads). The initial genome assemblies of E. crus-galli and E. oryzicola were from Ye et al., 2020. Genome annotations and resequencing data of other monocots were from NCBI and ENA.
Data analysis	(1) Genome assembly and annotation: Hifiasm (v0.12-r304), minimap2 (v2.15-r905), racon (v1.4.3), BWA (v0.7.15-r1140), AllHiC were used in assembly. Fgenesh, AUGUSTUS, EVidenceModeler (v1.1.1) and InterproScan (v5.24-63.0) were used in genome annotation.<br>(2) Comparative genomics and genome evolution: DAGchainer, KaKs_calculator, MAFFT (v7.310), RaxML, IQ-TREE (v1.6.12), ASTRAL (v5.7.4) and QuIBL were used.<br>(3) Population genomics: Bowtie2 and GATK pipeline were used in calling variants. FastTreeMP, Plink (v1.90b6.20) and fastStructure were used in population structure and phylogeny. NOVOplasty (v4.2) was used in chloroplast analysis.<br>(4) Demographic history was performed by est-sfs (v2.03), fastsimcoal2 and Stairway Plot(v2.0).<br>(5) Adaptation and selection analysis: VCFtools was used in calculating genetic indexes (nucleotide diversity, Fst, Tajima's D). GWAS was performed using EMMAX. PopLDdecay (v3.31) was used in linkage disequilibrium analysis.<br>The custom scripts used in this study have been deposited in the GitHub repository (https://github.com/bioinplant/Echinochloa_genome).

For manuscripts utilizing custom algorithms or software that are central to the research but not yet described in published literature, software must be made available to editors and reviewers. We strongly encourage code deposition in a community repository (e.g. GitHub). See the Nature Portfolio guidelines for submitting code & software for further information.

Data

Policy information about availability of data

All manuscripts must include a data availability statement. This statement should provide the following information, where applicable:

- Accession codes, unique identifiers, or web links for publicly available datasets
- A description of any restrictions on data availability
- For clinical datasets or third party data, please ensure that the statement adheres to our policy

The Echinochloa genome assemblies and annotations generated in this study are available at National Geophysical Data Center (NGDC) database (https://bigd.big.ac.cn) under the accession number PRJCA003883. The HiFi sequencing, Hi-C, and RNA-Seq data for genome assembly and annotation in this study have been deposited in NGDC (PRJCA003883). The raw data of 409 newly re-sequenced individuals are available at NGDC (PRJCA003883). The data of 328 previously re-sequenced barnyard grass accessions were retrieved from NGDC (accession number PRJCA001519). Besides Echinochloa genomes, other 36 monocot genomes used in this study were downloaded from EnsemblPlants (https://plants.ensembl.org) or National Center for Biotechnology Information (NCBI) GenBank (https://www.ncbi.nlm.nih.gov/).

Field-specific reporting

Please select the one below that is the best fit for your research. If you are not sure, read the appropriate sections before making your selection.

[x] Life sciences [ ] Behavioural & social sciences [ ] Ecological, evolutionary & environmental sciences

For a reference copy of the document with all sections, see nature.com/documents/nr-reporting-summary-flat.pdf

Life sciences study design

All studies must disclose on these points even when the disclosure is negative.

Sample size	737 Echinochloa samples were collected from 16 countries (China, Japan, Korea, Malaysia, India, and Pakistan in Asia; Brazil, US, Costa Rica, Uruguay, and Venezuela in America; Italy, Germany, Portugal, and Russia in Europe; Australia in Oceania), covering main rice-producing areas worldwide.
Data exclusions	No data were excluded from the analyses.
Replication	Eight individuals per accession were planted and phenotyped in 2017, 2018 and 2020 in CNRRI, China.
Randomization	This is not applicable as there is no experiments involved.
Blinding	All designs were transparent to every participants.

Reporting for specific materials, systems and methods

We require information from authors about some types of materials, experimental systems and methods used in many studies. Here, indicate whether each material, system or method listed is relevant to your study. If you are not sure if a list item applies to your research, read the appropriate section before selecting a response.

Materials & experimental systems

n/a	Involved in the study
[x]	[ ] Antibodies
[x]	[ ] Eukaryotic cell lines
[x]	[ ] Palaeontology and archaeology
[x]	[ ] Animals and other organisms
[x]	[ ] Human research participants
[x]	[ ] Clinical data
[x]	[ ] Dual use research of concern

Methods

n/a	Involved in the study
[x]	[ ] ChIP-seq
[x]	[ ] Flow cytometry
[x]	[ ] MRI-based neuroimaging
